# COUP-TFII Controls Mouse Pancreatic β-Cell Mass through GLP-1-β-Catenin Signaling Pathways

**DOI:** 10.1371/journal.pone.0030847

**Published:** 2012-01-24

**Authors:** Marie Boutant, Oscar Henrique Pereira Ramos, Cécile Tourrel-Cuzin, Jamileh Movassat, Anissa Ilias, David Vallois, Julien Planchais, Jean-Paul Pégorier, Frans Schuit, Patrice X. Petit, Pascale Bossard, Kathrin Maedler, Anne Grapin-Botton, Mireille Vasseur-Cognet

**Affiliations:** 1 Institute national del santé et de la recherché medicale(INSERM), Department of Endocrinology, Metabolism and Cancer, Cochin Institute, Paris-France; 2 Centre national de la recherche scientifique (CNRS), Paris, France; 3 Université Paris Descartes, Sorbonne Paris Cité, France; 4 Unit of Functional and Adaptative Biology, Laboratory of Biology and Pathology of the Endocrine Pancreas, Paris Diderot University, Paris, France; 5 Center for Integrative Genomics, University of Lausanne, Lausanne, Switzerland; 6 Department of Molecular Cellular Biology, Leuven, Belgium; 7 Centre national de la recherche scientifique (CNRS), Cochin Institute, Paris, France; 8 Ecole Polytechnique Fédérale de Lausanne, Lausanne, Switzerland; 9 Centre for Biomolecular Interactions Bremen, University of Bremen, Germany; Ecole Normale Supérieure de Lyon, France

## Abstract

**Background:**

The control of the functional pancreatic β-cell mass serves the key homeostatic function of releasing the right amount of insulin to keep blood sugar in the normal range. It is not fully understood though how β-cell mass is determined.

**Methodology/Principal Findings:**

Conditional chicken ovalbumin upstream promoter transcription factor II (COUP-TFII)-deficient mice were generated and crossed with mice expressing Cre under the control of pancreatic duodenal homeobox 1 (pdx1) gene promoter. Ablation of COUP-TFII in pancreas resulted in glucose intolerance. Beta-cell number was reduced at 1 day and 3 weeks postnatal. Together with a reduced number of insulin-containing cells in the ductal epithelium and normal β-cell proliferation and apoptosis, this suggests decreased β-cell differentiation in the neonatal period. By testing islets isolated from these mice and cultured β-cells with loss and gain of COUP-TFII function, we found that COUP-TFII induces the expression of the β-catenin gene and its target genes such as cyclin D1 and axin 2. Moreover, induction of these genes by glucagon-like peptide 1 (GLP-1) via β-catenin was impaired in absence of COUP-TFII. The expression of two other target genes of GLP-1 signaling, GLP-1R and PDX-1 was significantly lower in mutant islets compared to control islets, possibly contributing to reduced β-cell mass. Finally, we demonstrated that COUP-TFII expression was activated by the Wnt signaling-associated transcription factor TCF7L2 (T-cell factor 7-like 2) in human islets and rat β-cells providing a feedback loop.

**Conclusions/Significance:**

Our findings show that COUP-TFII is a novel component of the GLP-1 signaling cascade that increases β-cell number during the neonatal period. COUP-TFII is required for GLP-1 activation of the β-catenin-dependent pathway and its expression is under the control of TCF7L2.

## Introduction

Type 2 diabetes mellitus (T2DM) is a multifactorial disorder associated with impaired pancreatic β-cell function and insulin resistance. The onset of β-cell dysfunction in T2DM is complex, involving genetic and environmental factors that lead to decreased insulin secretion and reduced β-cell mass [1]. The identification of pathways that regulate β-cell function and mass in concert may lead to the development of novel therapeutic strategies for the treatment of T2DM and related disorders.

Chicken Ovalbumin Upstream Promoter Transcription Factor II (COUP-TFII, formerly known as NR2F2) belongs to the subfamily of nuclear hormone receptors that includes hepatocyte nuclear factor (HNF) 4α/maturity onset diabetes of the young (MODY)1 and the retinoid X receptor [2]. COUP-TFII acts in genetic programs associated with insulin biosynthesis and secretion in pancreatic β-cells, and in the regulation of lipid/energy metabolism and white adipose tissue development. Our original finding that COUP-TFII is highly expressed in islet β-cells [3], [4] led us to focus on the role of the COUP-TFII on pancreatic β-cell function. Based on evidence from an adult heterozygous β-cell COUP-TFII knockout mouse model and *ex vivo* and *in vitro* experiments, we reported that a decrease in COUP-TFII expression in β-cells is associated with defects in insulin production and secretion but β-cell mass is unaffected [3], [5]. Knockout mice with heterozygous deletion of COUP-TFII and mice with complete disruption of HNF4α in pancreatic β-cells have similar defects in insulin secretion, which led us to propose a model of transcriptional crosstalk between these two nuclear receptors. We then established that COUP-TFII contributes to the control of insulin secretion through the complex HNF4α/MODY1 transcription factor network operating in β-cells [4].

The well-characterized canonical Wnt/β-catenin pathway is critical for the development, renewal and function of various tissues. In the absence of Wnt, cytoplasmic β-catenin is unstable due to its phosphorylation and is degraded. Upon binding of Wnt to Frizzled receptor, which prevents β-catenin phosphorylation, the canonical pathway is activated. The unphosphorylated form of β-catenin accumulates in the cytoplasm, resulting in its stabilization and translocation into the nucleus. Once inside the nucleus, β-catenin, acting with transcription factors such as T-cell factor 7- like 2 (TCF7L2), stimulates transcription of various Wnt target genes, including cyclin D, c-myc and axin 2 [6], [7].

Recently, several gene loci that encode proteins that are either components of, or known target genes for the β-catenin/TCF7L2-dependent Wnt signaling pathway, have been discovered to confer susceptibility to the development of T2DM in human genetic studies. For example, at-risk alleles of TCF7L2 are associated with diabetic phenotypes characterized by impaired β-cell function and a reduction in GLP-1-induced potentiation of insulin secretion [8], [9], [10], [11]. The role of the canonical Wnt/β-catenin pathway in pancreatic β-cells is somewhat complex, because it differs depending on the age of the animal [12], [13], [14], [15] and crosstalk with other signaling pathways [16], [11]. The gluco-incretin hormone glucagon-like peptide 1 (GLP-1) or the long-acting agonist of the GLP-1 receptor (GLP-1R), exendin-4 (Exd4), activate β-catenin/TCF7L2 signaling to induce cyclin D1 and cell proliferation [17]. GLP-1 can also prevent apoptosis [18], [19], [20] and restore functional pancreatic β-cell mass in rodent models [21], [22], [23], [24].

Here, we report that COUP-TFII is a novel component of the GLP-1 signaling cascade that increases β-cell number during the neonatal period.

## Results

### Pdx1^CRE/-^ COUP-TFII^Fl/Fl^ mice are glucose intolerant

**Figure 1:**
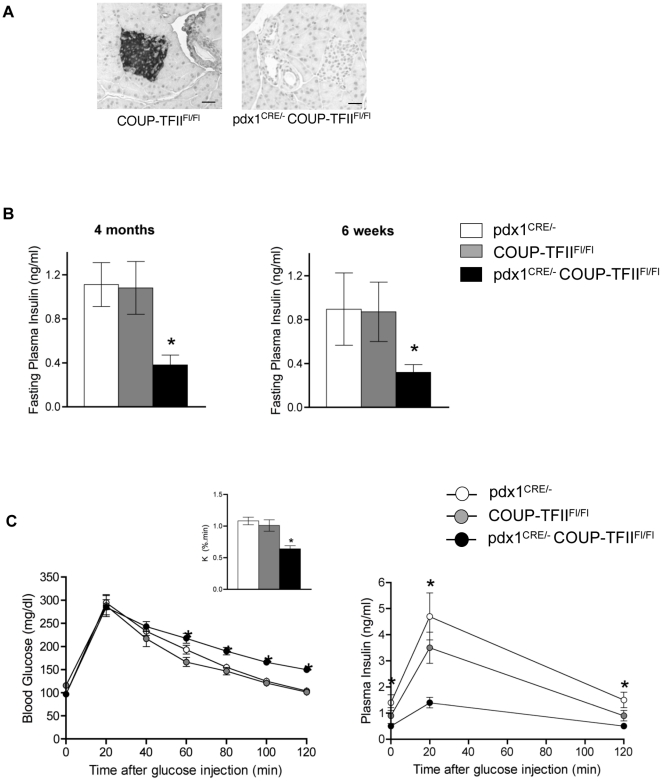
Pdx1^CRE/-^ COUP-TFII^Fl/Fl^ mice have defective glucose homeostasis. (A) Immunostaining of pancreatic sections from COUP-TFII^Fl/Fl^ control and pdx1^CRE/-^ COUP-TFII^Fl/Fl^ adult mice using mouse monoclonal antibodies against COUP-TFII. Scale bars =  100 µm (B) Fasting plasma insulin of 4 month and 6- week-old mice littermates. (C) Intraperitoneal glucose tolerance tests were performed in 4-month-old male pdx1^CRE/-^, COUP-TFII^Fl/Fl^ and pdx1^CRE/-^ COUP-TFII^Fl/Fl^ mice that had been fasted for 14 h. Blood glucose levels, left panel. Rate of glucose disappearance (K), inset panel. Plasma insulin levels, right panel. Results are mean ± SEM (error bars) for 8 to 14 animals per genotype. Values for pdx1^CRE/-^ COUP-TFII^Fl/Fl^ are significantly different from values for COUP-TFII^Fl/Fl^ and pdx1^CRE/-^ control mice at *P*<0.05 (*) or *P*<0.03 (**).

Since the nuclear receptor COUP-TFII is highly expressed in pancreatic β-cells, we generated mice to study the consequences of total β-cell ablation of COUP-TFII gene expression on β-cell function and glucose homeostasis using the Cre recombinase/loxP system. The pancreatic duodenal homeobox 1 (pdx1)**^CRE^** transgenic mouse line used in this study has a robust and early Cre recombinase activity in pancreas progenitors, in exocrine (acinar and ductal) cells and endocrine cells during development and after birth is active only in pancreatic β-cells [25]. Pdx1**^CRE/-^** COUP-TFII**^Fl/Fl^** mutant mice were generated by mating COUP-TFII **^Fl/wt^** with pdx1**^CRE/-^** COUP-TFII **^Fl/wt^** and their phenotypes were compared with controls pdx1**^CRE/-^** and COUP-TFII**^Fl/Fl^**. As previously reported, immunostaining with COUP-TFII antibodies showed COUP-TFII protein present in pancreatic β-cells of control COUP-TFII**^Fl/Fl^** or pdx1**^CRE/-^** mice [4], but not in pancreatic β-cells of pdx1**^CRE/-^** COUP-TFII**^Fl/Fl^** mice (Fig. 1A). We did not detect COUP-TFII expression in mouse enteroendocrine cells in the duodenum where pdx1 expression had been reported [26] (data not shown).

Both male and female pdx1**^CRE/-^** COUP-TFII**^Fl/Fl^** mice had very low fasting serum insulin levels compared to control littermates at four months of age (Fig. 1B, left panel). This fasting hypoinsulinemia was already detected in six-week-old mice (Fig. 1B right panel). When glucose was administered in a glucose tolerance test, all three groups of adult male mice showed very similar blood glucose levels after 20 minutes (Fig. 1C left panel). From 60 minutes onwards, pdx1**^CRE/-^** COUP-TFII**^Fl/Fl^** mice had higher blood glucose levels than pdx1**^CRE/-^** and COUP-TFII**^Fl/Fl^** control littermates (Fig. 1C left panel). In addition, the glucose disappearance rate (K) in pdx1**^CRE/-^** COUP-TFII**^Fl/Fl^** mice was almost half of that in control littermates (Fig. 1C), confirming that mutant mice were glucose intolerant. Insulin levels were significantly lower in pdx1**^CRE/-^** COUP-TFII**^Fl/Fl^** mice than in controls at all corresponding time points, additional evidence that the knockout mice have a defect in insulin secretion in response to glucose (Fig. 1C right panel). Similar results were obtained with female mice (data not shown). The combination of glucose intolerance and inappropriate low insulin levels during the glucose tolerance test led us to further examine the functional β-cell mass in pdx1**^CRE/-^** COUP-TFII**^Fl/Fl^** mice.

### Pdx1^CRE/-^ COUP-TFII^Fl/Fl^ mice have reduced pancreatic β-cell mass at the neonatal stage

**Figure 2:**
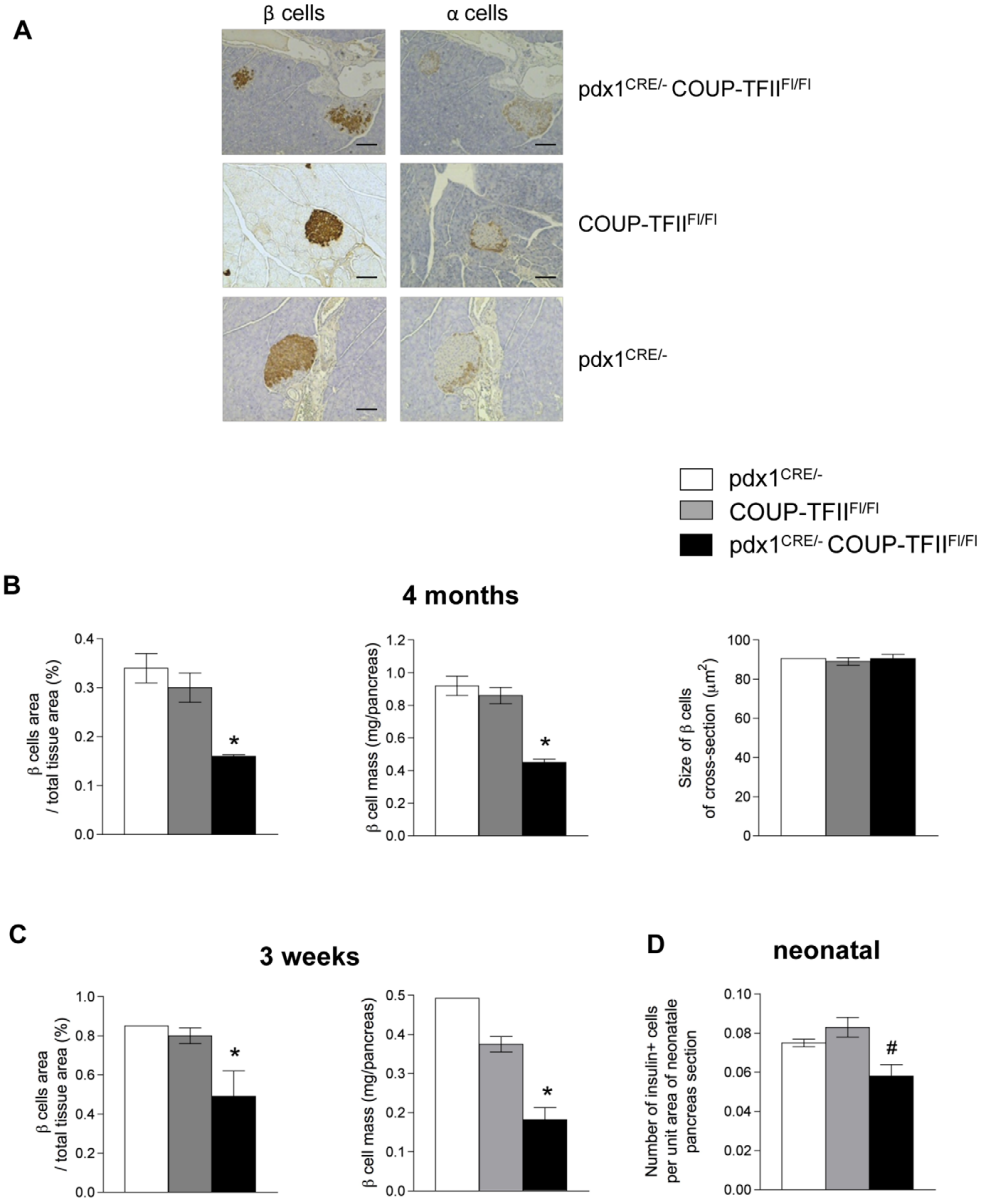
Pdx1^CRE/-^ COUP-TFII^Fl/Fl^ mice have fewer β-cells at birth. (A) Pancreatic sections from adult mice were immunostained for pancreatic β-cells and α-cells using respectively anti-insulin and anti-glucagon antibodies. Scale bars =  100 µm (B) Estimation of the relative β-cell area and β-cell mass in the pancreatic sections of pdx1^CRE/-^, COUP-TFII^Fl/Fl^ and pdx1^CRE/-^COUP-TFII^Fl/Fl^ in 4-month-old mice. Beta-cell mass was estimated by morphometric analysis. Relative β-cell size (mean of ≥ 1500 cells counted). (C) Estimation of β-cell number and β-cell mass in pancreatic sections of 3-week-old mice. (D) Ratio of Insulin-positive cross-sectional area relative to pancreas (DAPI-positive) cross-sectional area in one-day-old neonate animals. # The reduction of pdx1^CRE/-^, COUP-TFII^Fl/Fl^ over the cumulated controls (pdx1^CRE/-^ and COUP-TFII^Fl/Fl^) is already quite important (23%) and is significant with student *t*-test and non-parametric tests (2-tailed Mann-Whitney U test, which does not assume normal distribution: p = 0.0476). It is not significant if referred individually to the two control populations. Data are means ± SEM (error bars) of values from 4 animals of each genotype. *Values for pdx1^CRE/-^, COUP-TFII^Fl/Fl^ mice is significantly different from the corresponding values for control mice (pdx1^CRE/-^ and COUP-TFII^Fl/Fl^) at *P*<0.05.

Whole pancreases from adult pdx1**^CRE/-^** COUP-TFII**^Fl/Fl^** mice weighed 281 ± 5.5 mg, similar to pancreases from COUP-TFII**^Fl/Fl^** weighing 291 ± 12 mg and from pdx1**^CRE/-^** weighing 271.5 ± 1.5 mg. Islets from adult mice were immunostained for insulin, indicative of β-cells, and for glucagon, indicative of α-cells. Islets from adult pdx1**^CRE/-^** COUP-TFII**^Fl/Fl^** mice had a typical tissue arrangement where the majority of cells, insulin-positive β-cells, were surrounded by fewer glucagon-positive α-cells at the periphery (Fig. 2A). There were fewer β-cells in the islet core in pdx1**^CRE/-^** COUP-TFII**^Fl/Fl^** mice than in control mice. Quantitative analysis of the cellular composition of pancreatic sections showed that the fraction of β-cells was 50% smaller in pdx1**^CRE/-^** COUP-TFII**^Fl/Fl^** (Fig. 2B) indicating a decrease in β-cell mass (Fig. 2B). In contrast, α-cell fraction and α-cell mass in pdx1**^CRE/-^** COUP-TFII**^Fl/Fl^** animals were similar to those found in control groups (Figure S1A). An overall decrease in β-cell mass could result either from smaller β-cells (hypotrophy) or fewer β-cells (hypoplasia). To estimate β-cell size, we measured the cross-sectional area of individual β-cells in pancreas sections. The average cross-sectional area of individual β-cells in pdx1**^CRE/-^** COUP-TFII**^Fl/Fl^** mice was not different from controls supporting the notion that the decrease in the total β-cell mass is probably due to β-cell hypoplasia (Fig. 2B). A similar decrease of 50% in β-cell number was measured in pdx1**^CRE/-^** COUP-TFII**^Fl/Fl^** mice at 3 weeks of age (Fig. 2C). At postnatal day 1(P1), we observed β-cell hypoplasia with a 23% reduction in β-cells compared to littermate controls (Fig. 2D). Since Pdx1-Cre is expressed in the pancreatic epithelium during embryogenesis [25] and COUP-TFII expression can be detected in insulin-positive embryonic cells [27], we counted the endocrine cells present at E12.5 and E14.5. The total numbers of glucagon- and insulin-expressing cells were similar in mutant mice and in control littermates at both stages (Figure S1B and Figure S1C). Therefore, lower insulin secretion and impaired regulation of glucose homeostasis in pdx1**^CRE/-^** COUP-TFII**^Fl/Fl^** mice is most likely the result of a decrease in β-cell number that occurs at the end of gestation and around birth.

### In vivo COUP-TFII ablation decreases pancreatic β-cell differentiation

**Figure 3:**
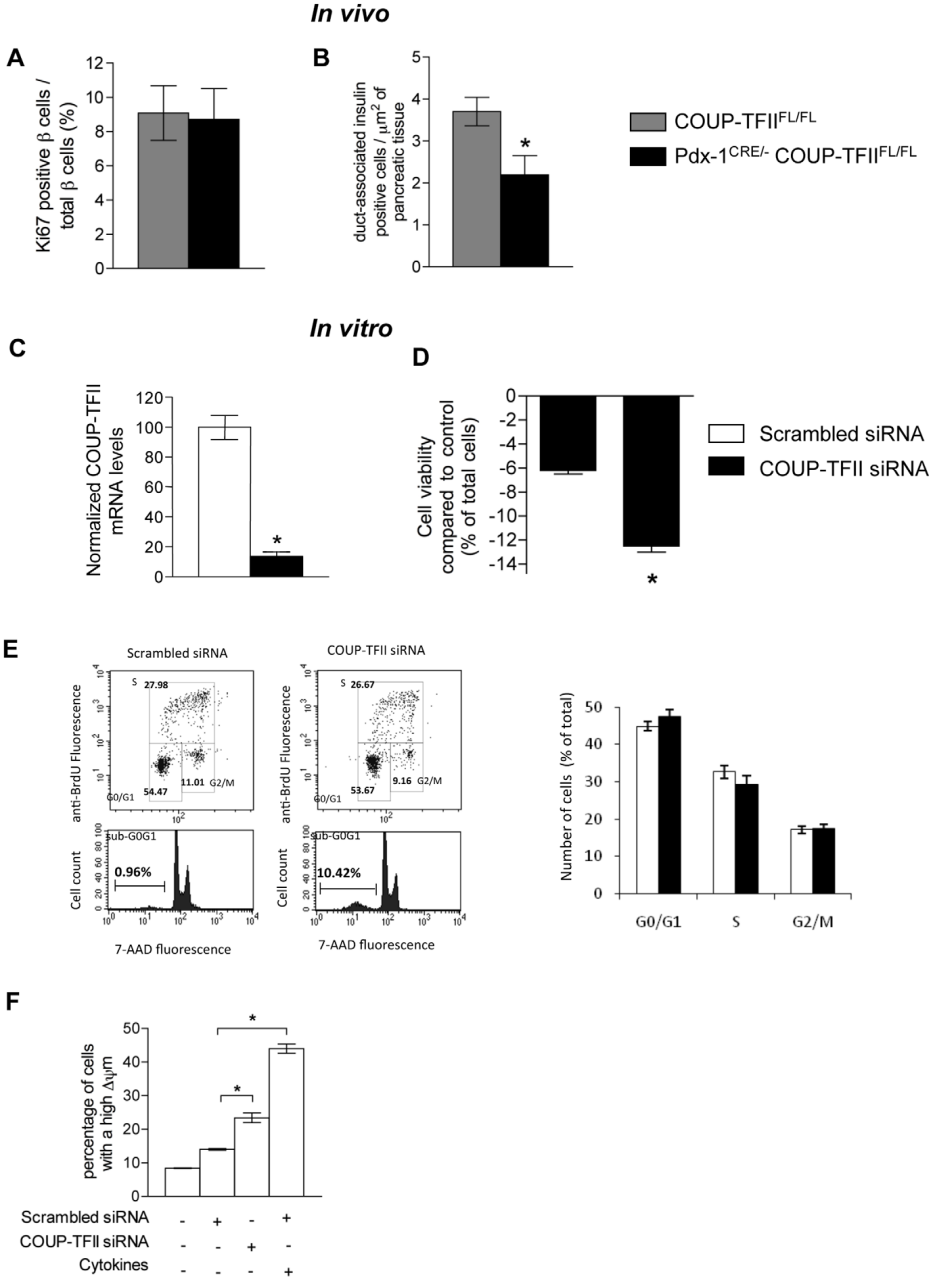
Five-day-old pdx1^CRE/-^, COUP-TFII^Fl/Fl^ mice have reduction of β-cell neogenesis and COUP-TFII knockdown increases 832/13 INS-1 β-cells apoptosis. In pancreatic sections of 5-day-old mice (A) β-cell proliferation was assessed by double staining insulin/Ki67 (B) β-cell neogenesis activation was evaluated through quantification of duct-associated insulin+ β-cells per unit of total tissue area. 832/13 INS-1 β-cells were electroporated with scrambled or specific COUP-TFII siRNA and cultured in INS-1 medium for 24 h or 48 h. (C) Relative COUP-TFII mRNA levels determined by RT-qPCR at 48 h. (D) Effect of COUP-TFII siRNA on cell viability analyzed by MTT assay (n = 5 electroporations). (E) Effect of COUP-TFII siRNA on β-cell proliferation. 832/13 INS-1 β-cells were cultured in INS-1 medium for 48 h and stained with BrdU. Cells were analyzed by flow cytometry (n = 4 electroprations). (F) Effect of COUP-TFII siRNA on β-cells apoptosis and comparison of the apoptotic rate with cell treated with a rat cytokine mix containing 25 ng/ml TNF-α, 10 ng/ml IL-1β and 10 ng/ml INF-γ during 24 h. 832/13 INS-1 β-cells were electroporated with scrambled or COUP-TFII siRNA and 48 h later they were stained with DiOC6(3) and analyzed by flow cytometry (n = 4 electroporations). * Significant difference between values linked by brackets at *P*<0.03.

Beta-cell mass is increased by β-cell proliferation, β-cell hypertrophy (increased cell size), and β-cell neogenesis (differentiation from duct cells) and is decreased by β-cell death primarily through apoptosis [28]. We sought to determine which mechanisms *in vivo* and *in vitro* led to the observed decrease in β-cell mass. In *vivo,* we evaluated the proliferating β-cells by double staining of pancreatic sections for insulin and the G1, S, G2 and M marker Ki67 (Fig. 3A). We found that COUP-TFII ablation did not alter β-cell proliferation in the pancreas in 5-day-old (P5) pdx1**^CRE/-^** COUP-TFII**^Fl/Fl^** mice compared to the COUP-TFII**^Fl/Fl^** control mice. We then looked at apoptotic cells within these pancreases. In all groups, the frequency of β-cell apoptosis was extremely low and we were unable to detect any differences between genotypes at this stage (data not shown). Through lineage tracing experiments it has recently been shown that β-cell neogenesis from ducts still occurs at P1 [29], [30]. We next evaluated an intermediate step of β-cell differentiation by measuring the number of small clusters of insulin containing cells within the ductal epithelium at P5. Cluster number was significantly lower in the pancreas of pdx1**^CRE/-^** COUP-TFII**^Fl/Fl^** compared to control (Fig. 3B). These results together with the reduced number of β-cells at P1 and further reduction at 3 weeks suggest a decrease in neogenesis at the end of gestation and during the neonatal period.

We next examined the effect of COUP-TFII knockdown on the viability of 832/13 INS-1 β-cells in culture. As already reported [4], electroporation of a specific-COUP-TFII siRNA into 832/13 INS-1 cells causes a 80% decrease in COUP-TFII levels (Fig. 3C). Cell viability was determined here by two approaches. Firstly, in the colorimetric MTT assay, there were fewer living cells at 24 h and significantly fewer at 48 h in COUP-TFII siRNA-transfected 832/13 INS-1 cells compared to scrambled siRNA-transfected control cells (Fig. 3D). Secondly, we used flow cytometry FACS analysis to examine the effect of COUP-TFII on cell number, proliferation and apoptosis. Staining with BrdU revealed that both cell populations underwent normal cell cycle progression confirming our *in vivo* observations. However, a sub G0/G1 peak, a hallmark of apoptotic cells, was detected in the COUP-TFII siRNA-transfected 832/13 INS-1 cell populations but not in scrambled siRNA-transfected control populations (Fig. 3E). Since a drop in mitochondrial membrane potential (Δψm) is one of the first events in early apoptosis [31], we measured Δψm in transfected cells. As shown in figure 3F, the number of cells with a large Δψm doubled in the COUP-TFII knockdown cell population indicating a 2-fold increase in apoptotic rate compared to the control cell population. This degree of apoptosis induction is comparable to the effect of high concentrations of cytokines, known inducers of β-cell apoptosis [20], used here as a positive control that induced a 4-fold increase in apoptosis. Altogether, *in vitro*, these results suggest that COUP-TFII maintains β-cell survival.

### COUP-TFII induces expression of β-catenin and its target genes in pancreatic β-cells

**Figure 4:**
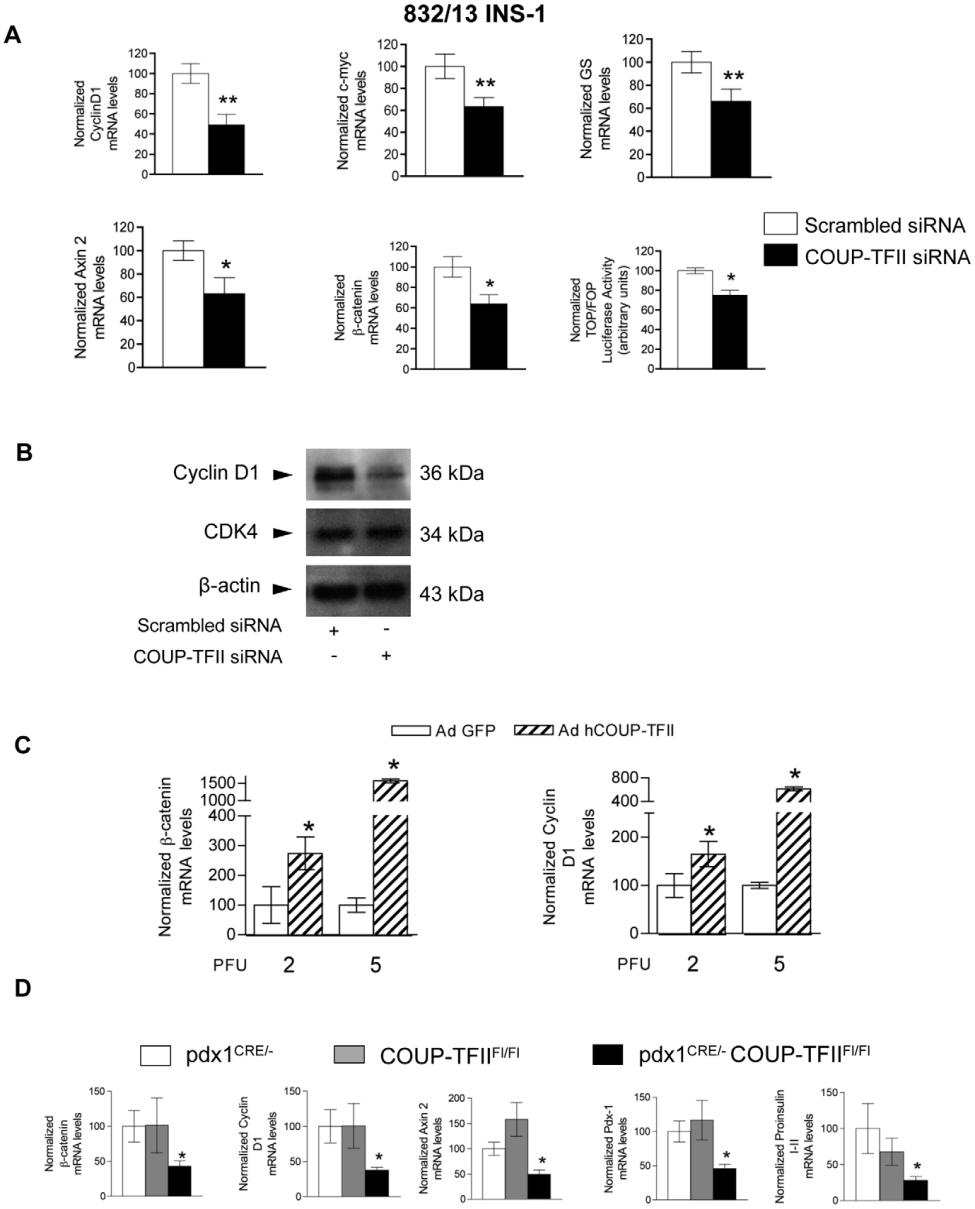
COUP-TFII regulates β-catenin signaling target genes in mouse islets and a β-cell line. (A) 832/13 INS-1 β-cells were electroporated with scrambled (control) or COUP-TFII specific siRNA and cultured for 48 h. Levels of cyclin D1, c-myc, glutamine synthase, axin 2, and β-catenin mRNA were determined by RT-qPCR. Effects on Wnt/β-catenin signaling were assessed by the luciferase activity of a TOP/FOP-flash reporter. Results are means ± SEM of data from between 3 and 12 (mRNA levels) independent electroporation experiments. Significant differences between COUP-TFII knockdown and control values are shown by asterisks at the *P* value indicated. *P<0.05; **P<0.03. (B) A representative immunoblot of different experiments of 832/13 INS-1 β-cells that were electroporated with scrambled or COUP-TFII specific siRNA. 50 µ g of total protein extract was subjected to immunoblot analysis with antibodies against cyclin D1, CDK4 or β-actin. (C) RT-qPCR analysis of β-catenin and cyclin D1 mRNA levels of 832/13 INS-1 cells infected with Ad-GFP or Ad-hCOUP-TFII at 2 or 5 PFU per cell as indicated [4] normalized to housekeeping gene cyclophilin. Results are means ± SEM of data from 3 independent infection experiments. *Significant difference between between COUP-TFII overexpression and control values at *P*<0.05 (D) RT-qPCR analysis of β-catenin, cyclin D1, axin 2, pdx1 and proinsulin I-II mRNA from islets from 5-week-old pdx1^CRE/-^, COUP-TFII^Fl/Fl^ and pdx1^CRE/-^ COUP-TFII^Fl/Fl^ mice. Results are mean ± SEM (error bars) for 5 animals per genotype. *P<0.05 for pdx1^CRE/-^ COUP-TFII^Fl/Fl^ versus COUP-TFII^Fl/Fl^ and pdx1^CRE/-^ control mice.

To understand the molecular mechanisms by which COUP-TFII controls β-cell mass, we determined the RNA profile of COUP-TFII knockdown β-cells using Affymetrix oligonucleotide microarrays. This microarray expression analysis identified 464 significantly up-regulated and 505 significantly down-regulated genes in COUP-TFII knockdown β-cells (GEO). Computational analysis identified clusters of genes shared by different gene regulatory networks (Figure S2 and Figure S3) namely genes involved in Wnt/β-catenin signaling, insulin signaling and lipid/carbohydrate metabolism. Cyclin D1 is a well-characterized direct target gene of β-catenin/TCF7L2-dependent Wnt signaling in β-cells [17]. COUP-TFII coordinates cell patterning through modulation of Wnt signaling in other systems [32], [33]. Therefore, we examined whether knockdown of COUP-TFII in 832/13 INS-1 cells modulates β-catenin/TCF7L2-dependent Wnt targets. Our microarray analysis showed that levels of the cyclin D1 (Ccnd1) transcripts were lower in COUP-TFII depleted cells than in control cells (GEO database, accession number: GSE30526). As shown in figure 4A, independent RT-qPCR experiments indicated that cyclin D1 mRNA levels were lowered by 55% in COUP-TFII depleted cells. Immunoblot analysis indicated that cyclin D1 protein levels also fell coinciding with observed changes in mRNA levels (Fig. 4B). However, protein levels of the cyclin D-dependent kinase 4 (cdk-4), a kinase associated with cyclin D1 in controlling G1 phase of the cell cycle, were not modified in COUP-TFII knockdown β-cells (Fig. 4B). Messenger RNA levels of Wnt target genes c-myc, glutamine synthase and axin 2 all significantly decreased in COUP-TFII depleted 832/13 INS-1 cells compared to control cells (Fig. 4A) whereas cyclin D2 mRNA levels were not affected (data not shown). We also examined whether COUP-TFII, being a transcription factor, could control the expression of β-catenin itself under the same experimental conditions. Indeed, knockdown of COUP-TFII caused a significant 36% decrease in β-catenin mRNA levels (Fig. 4A). To further address the relationships between COUP-TFII and β-catenin/TCF7L2-dependent Wnt signaling, we transfected COUP-TFII depleted 832/13 INS-1 cells with the TOP/FOPflash luciferase vectors, a TCF/β-catenin-directed transcription system in which activity of the luciferase reporter indicates activation of signaling via β-catenin. TCF/β-catenin-dependent luciferase activity decreased significantly by 25% in the absence of COUP-TFII (Fig. 4A). In contrast, adenovirus-mediated overexpression of human COUP-TFII in 832/13 INS-1 cells induced both β-catenin and cyclin D1 mRNA levels (Fig. 4C).

We next examined the Wnt signalling pathways in islets isolated from 5-week-old mutant pdx1**^CRE/-^** COUP-TFII**^Fl/Fl^** mice. Messager RNA levels of β-catenin, cyclin D1 and axin 2 were markedly lower than in control islets (Fig. 4D). We also examined the expression of pdx1, a master regulator of functional pancreatic β-cell mass, and one of its target genes, proinsulin I-II. We found that mRNA levels of both of these genes were significantly decreased in COUP-TFII-ablated islets of pdx1**^CRE/-^** COUP-TFII**^Fl/Fl^** mice compared to those from control littermates (Fig. 4D). This reduction of pdx1- expression is probably a consequence of the absence of COUP-TFII, not of direct control [5]. Collectively, these results indicate that there is crosstalk between COUP-TFII and the Wnt signaling pathway which may promote pancreatic β-cell differentiation.

### COUP-TFII is required for GLP-1 induced activation of the β-catenin/TCF7L2 axes and its target gene cyclin D1

**Figure 5:**
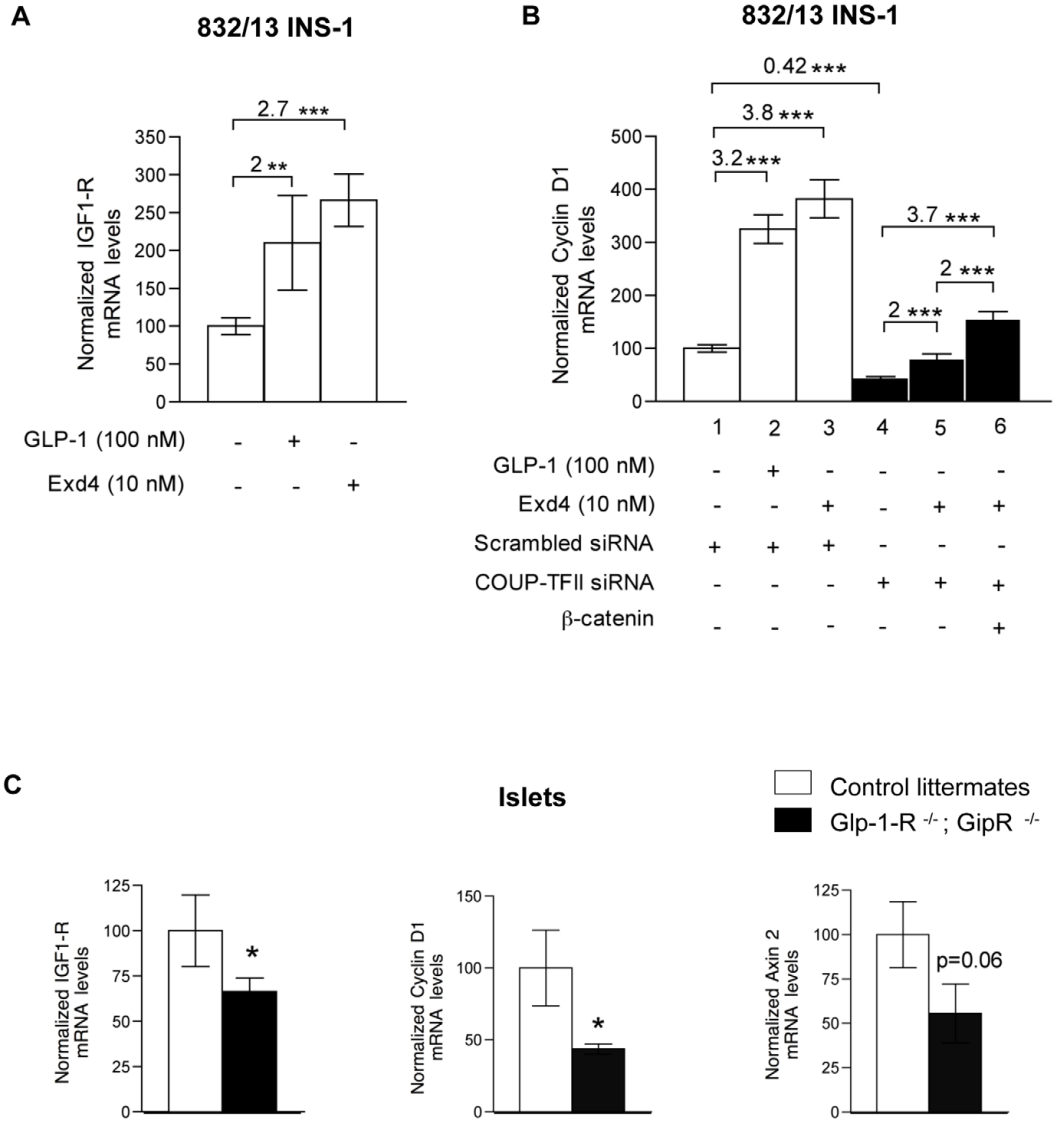
GLP-1 signaling enhances cyclin D1 gene expression via COUP-TFII and β-catenin expression. (A) GLP-1 and Exd4 stimulate IGF1-R expression in the 832/13 INS-1 β-cells. Wild-type 832/13 INS-1 β-cells were cultured in INS-1 medium and stimulated for 6 h with (+) or without (-) 100 nM GLP-1 or 10 nM Exd4 and IGF1-R mRNA levels were analyzed by RT-qPCR and normalized to housekeeping gene cyclophilin. Numbers over brackets indicate fold increase between Exd4 and GLP-1 treated samples and the control as indicated. Results are means ± SEM of data from 5 independent electroporation experiments. Significant differences between values linked by brackets are shown by asterisks at *P* value indicated. **P<0.03. ***P<0.001. (B) Absence of COUP-TFII abrogates Exd4 stimulated cyclin D1 expression in 832/13 INS-1 β-cells. 832/13 INS-1 β-cells were electroporated with scrambled or specific COUP-TFII siRNA, infected or not infected with recombinant β-catenin adenovirus and stimulated with (+) or without (-) 100 nM GLP-1 or 10 nM Exd4 for 6 h. Cyclin D1 mRNA levels was analysed by RT-qPCR. Numbers indicate fold-increase of cyclin D1 expression. Results are means ± SEM of data from 5 independent electroporation experiments. ***Significant differences between values linked by brackets at *P*<0.001. (C) *In vivo* crosstalk of GLP-1 and Wnt signaling pathways. RT-qPCR analysis of IGF-1R, cyclin D1 and axin 2 mRNA levels were performed on mouse islets isolated from *GipR* ^-/-^; *Glp-1-R^-/-^* transgenic mice normalized to housekeeping gene cyclophilin. Data are means ± SEM for 5 animals of each genotype. *Significant difference between transgenic and control values at *P*<0.05.

The transcription of cyclin D1 is induced by exendin-4 (Exd4), a stable agonist of the glucagon-like peptide-1 receptor (GLP-1R), via TCF7L2 and β-catenin in INS-1 cells [17]. This prompted us to test whether COUP-TFII has a role in this process. We first verified that GLP-1 signaling occurs by measuring expression of insulin growth factor-1 receptor (IGF-1R), a known target gene of gluco-incretin hormone signaling [20] in 832/13 INS-1 cells. GLP-1 or Exd4 induced IGF-1R mRNA expression in cells within 6 h (Fig. 5A). Treatment with GLP-1 or Exd4 also induced cyclin D1 mRNA levels (Figure 5B lane 1 vs. lanes 2 and 3) in β-cells culture. As expected and as shown in figure 4A, transfection with COUP-TFII siRNA caused a decrease in basal cyclin D1 mRNA levels (Fig. 5B lane 1 vs. lane 4). In addition, COUP-TFII knockdown almost halved Exd4 induced increase in cyclin D1 mRNA (Fig. 5B lanes 1 and 3 vs. lanes 4 and 5). We then examined whether β-catenin is required for Exd4-mediated cyclin D1 induction, as might be expected, in wild type (Figure S4) and COUP-TFII depleted cells. This was done by adenovirus-mediated overexpression of β-catenin. In the absence of COUP-TFII, a 3.7 fold increase in cyclin D1 mRNA levels was restored when β-catenin was overexpressed (Figure 5B, lanes 4 and 6). These results demonstrate the requirement of COUP-TFII in the induction of cyclin D1 by GLP-1 via increased β-catenin levels.

Since GLP-1 is known to activate Wnt signaling in cultured β-cells via the GLP-1R [17], we compared the expression of some Wnt target genes in islets isolated from mice in which incretin receptors were inactivated. As previously reported [20], we found significantly lower IGF-1R mRNA levels in islets isolated from these GipR^-/-^; Glp-1R^-/-^ mice compared to control littermates. We also detected a marked decrease in cyclin D1 mRNA and axin 2 mRNA levels (Fig. 5C).

Altogether, these *ex vivo* and *in vivo* results indicate that full activation of the β-catenin/TCF7L2-dependent Wnt signaling pathway by GLP-1 via GLP-1R requires COUP-TFII expression in pancreatic β-cells.

### Absence of COUP-TFII in islets results in decreased GLP-1 receptor expression

**Figure 6:**
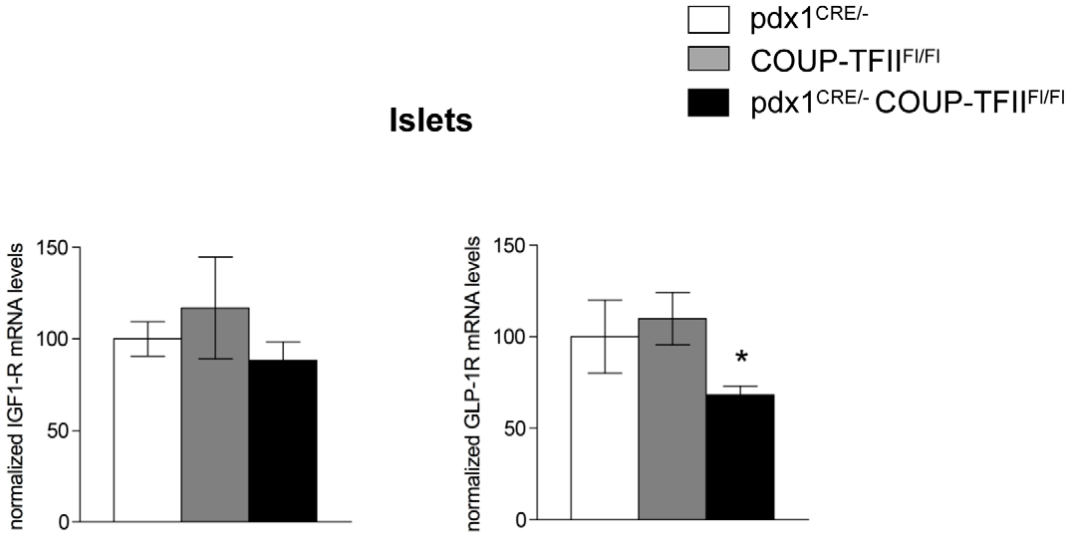
COUP-TFII controls GLP-1R expression in islet. RT-qPCR analysis of IGF-1R and GLP-1R mRNA from islets from 5-week-old pdx1^CRE/-^, COUP-TFII^Fl/Fl^ and pdx1^CRE/-^ COUP-TFII^Fl/Fl^ mice normalized to housekeeping gene cyclophilin. Results are mean ± SEM (error bars) for 5 animals per genotype. *P<0.05 for pdx1^CRE/-^ COUP-TFII^Fl/Fl^ versus COUP-TFII^Fl/Fl^ and pdx1^CRE/-^ control mice.

While the fold induction of cyclin D1 expression in response to exendin-4 recovers in COUP-TFII depleted 832/13 INS-1-cells when β-catenin is overexpressed, the absolute amount of cyclin D1 mRNA does not reach that of the control (Fig. 5B). It is likely that other components of the GLP-1 signaling pathway altered in the absence of COUP-TFII also contribute to the observed phenotype. We analyzed whether IGF-1R [20] and GLP-1R [34], two target genes of the GLP-1 signaling cascades, but not known to be β−catenin dependent targets, may be altered in islets isolated from 5-week-old mutant pdx1**^CRE/-^** COUP-TFII**^Fl/Fl^** mice. We did not detect any changes in IGF-1R mRNA levels but a significant reduction of GLP-1R mRNA levels were measured in islets isolated from 5-week-old mutant pdx1**^CRE/-^** COUP-TFII**^Fl/Fl^** mice compared to those from control littermates (Fig. 6).

### COUP-TFII expression is activated by TCF7L2 in β-cells

**Figure 7:**
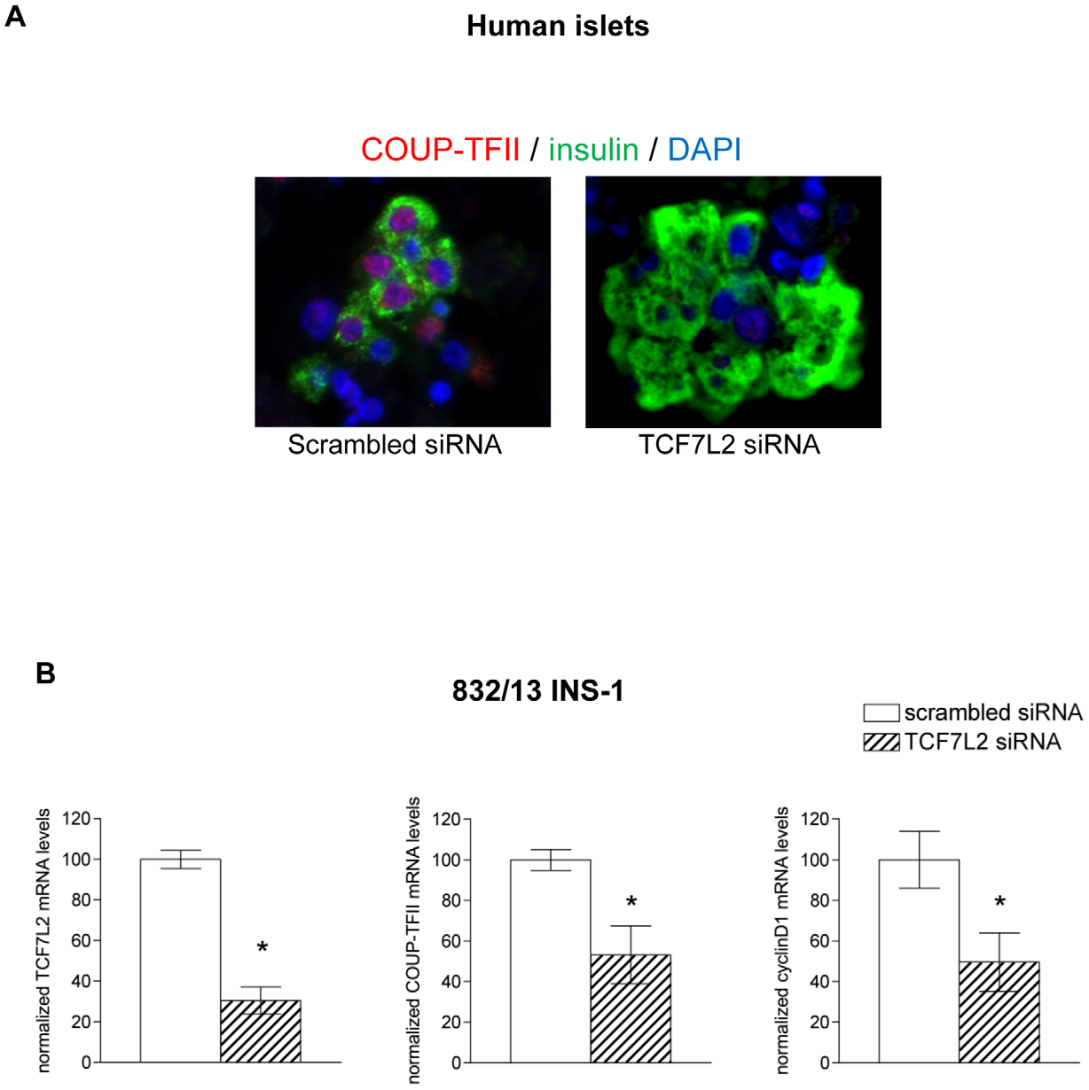
COUP-TFII expression is activated by TCF7L2 in β-cells. (**A**) Fixed healthy human islets were triple-stained for COUP-TFII in red, for insulin in green and DAPI in blue. (B) 832/13 INS-1 β-cells were electroporated with scrambled (control) or specific TCF7L2 siRNA and cultured for 48 h. Relative TCF7L2, COUP-TFII and cyclin D1 mRNA levels were determined by RT-qPCR normalized to housekeeping gene cyclophilin. Results are means ± SEM of data from between 4 independent electroporation experiments. Significant differences between TCF7L2 knockdown and control values are shown by asterisks at the *P* value indicated. *P<0.05.

Since there is a direct interplay between GLP-1R/GIP-R and TCF7L2 in human islets [34], we examined COUP-TFII protein expression in isolated human pancreatic islets exposed to siRNA directed to TCF7L2 or scrambled control siRNA and cultured as previously described [34]. Triple staining for COUP-TFII, insulin and DAPI revealed that COUP-TFII was almost undetectable in TCF7L2 depleted β-cells (Fig. 7A). Also down-regulation of TCF7L2 by TCF7L2 siRNA in rat 832/13 INS-1 resulted in down-regulation of COUP-TFII mRNA abundance and consequently decreased cyclin D1 expression when compared to scrambled siRNA. This control may represent a feedforward loop by which both COUP-TFII and TCF7L2 positively regulate each other.

## Discussion

The development and maintenance of pancreatic β-cell mass and function are important determinants of an organism's homeostatic capacity to respond to changing blood glucose levels effectively. In our previous study, we reported that when expression of the nuclear receptor COUP-TFII was depleted in pancreatic β-cells, adult RIP^cre/-^ COUP-TFII ^fl/wt^ mice were glucose intolerant due to impaired glucose-stimulated insulin secretion but β-cell mass at 14 weeks of age was unaffected [3]. Here we found that COUP-TFII is a novel positive regulator of β-cell mass during the neonatal period in mice.

In this study, we demonstrate the physiological and molecular consequences of homozygous COUP-TFII ablation selectively in pancreas using the pdx-1 promoter controlling Cre recombinase expression. We show that adult mice developed glucose intolerance due to a reduction in β-cell number at birth. The total number of glucagon- and insulin-expressing cells were similar in mutant mice and control littermates from E12.5 to E14.5 suggesting that COUP-TFII is not involved in the early development of endocrine cells. However, the reduction of the number of small clusters of insulin containing cells within the ductal epithelium at P5 together with the reduced number of β-cells at P1 and further reduction at 3 weeks suggests decreased neogenesis in the neonatal period. In addition, our results *in vitro* show that COUP-TFII expression maintained β-cell survival. Although we were unable to measure β-cell apoptosis *in vivo* due to very low numbers of cells as found previously [35], we cannot totally exclude the possibility that it contributes to β-cell reduction in COUP-TFII knockout mice.

Our molecular studies in islets isolated from 6-week-old pdx1**^CRE/-^** COUP-TFII**^Fl/Fl^** and in cultured INS-1 cells with loss and gain of COUP-TFII function provide the first evidence that this nuclear receptor participates in the Wnt/β-catenin/TCF7L2 dependent transcription signaling pathway in pancreatic β-cells. We show that COUP-TFII positively controls a TCF-sensitive luciferase reporter gene that is specifically triggered by Wnt signaling. We also observed that COUP-TFII increases β-catenin gene expression, a central component of the canonical Wnt-signaling pathway and consequently up-regulates some of the target genes in β-cells. Whether or not the effect on β-catenin transcription is mediated by direct COUP-TFII binding or by an indirect action through other binding partners is not yet known. Both mechanisms have been shown to be relevant to COUP-TFII action in other cell types [36]. For example, mouse IGF-1 and neuropilin-2 genes are positive direct targets of COUP-TFII [37], [38] and COUP-TFII binds to its promoter to negatively control its expression in pancreatic β-cells [4]. During the neonatal period, active Wnt/β-catenin-signaling is involved in the regulation of pancreatic β-cell number in normal and stressed rats mainly through proliferation and neogenesis [7]. In parallel TCF7L2 depletion in islets results in increased β-cell apoptosis and decreased β-cell proliferation [34].

**Figure 8:**
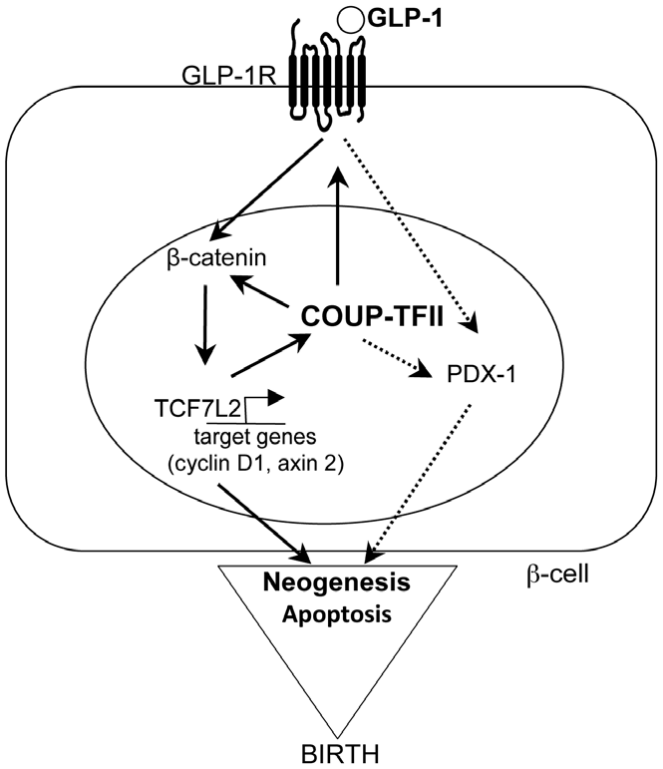
Proposed scheme of how COUP-TFII increases β-cell numbers through GLP-1 signaling cascade during the neonatal period in mice. Positive regulation (arrows) of indirect COUP-TFII target's genes in pancreatic β-cells.

Several hormones and growth factors activate the canonical Wnt signaling pathway [16], [11]. One such factor is GLP-1, which is a gut-derived incretin hormone [39]. GLP-1 induces multiple signaling pathways intrinsic to β-cell function [40], [41], [42]. GLP-1 protects pancreatic β-cells from apoptosis [20], [19] and promotes β-cell neogenesis in many rodent model systems and human pancreatic ducts [43]. In unstressed rodents, an exogenous GLP-1R agonist stimulates islet neogenesis [44] and restores a functional pancreatic β-cell mass in the newborn Goto-Kakizaki prediabetic rat [23]. A functional link between Wnt/β-catenin signaling and GLP-1 signaling has been established where GLP-1 boosts pancreatic β-cell division by activating TCF7L2-dependent Wnt signaling [17]. In human islets functional GLP1 signaling requires TCF7L2 [45]. Our results suggest that COUP-TFII acts as a molecular link between these two pathways. Our results demonstrate that COUP-TFII is required for GLP-1 activation of the β-catenin/TCF7L2 signaling pathway and its downstream target cyclin D in isolated islets and in INS-1 β-cells. In parallel, two target genes of GLP-1 signaling, GLP-1R and pdx-1 expression were significantly lower in mutant islets compared to control islets and COUP-TFII expression was lowered by depletion of the Wnt signaling-associated transcription factor TCF7L2 in human islets and rat β-cells. Altogether, these observations suggest a complex interplay between several components of the GLP-1 signal transduction pathway and we propose that COUP-TFII is possibly a link between TCF7L2 and GLP-1R expression (Figure 8).

In other tissues, COUP-TFII is involved in apoptosis, proliferation and differentiation [37], [38], [46], [47]. Our results suggest that COUP-TFII is a critical transcription factor for the expansion of β-cells during early neonatal life and COUP-TFII might participate in determining β-cell survival. Questions as to the relative contribution of β-cell proliferation versus islet neogenesis to β-cell mass expansion and which progenitor cells which are capable of generating endocrine cells during neonatal are highly controversial. There is evidence that GLP-1 can stimulate differentiation of β-cell precursors [48], [43] and prevent apoptosis [18], [19], [20]. We also observed expression of COUP-TFII in ductal cells at E18.5 (Figure S5) and at least a subset of ductal cells can still differentiate into endocrine cells in late development and the early post-natal period P1 [29], [30].

In conclusion, transcriptional control by COUP-TFII defines a novel pathway linking physiological insulinotropic signaling to mechanisms determining β-cell number in the pancreas. While there are many drugs already available for the treatment of T2DM, over two-thirds of people with the disease continue to have suboptimal glucose control, and incretin therapies offer benefits matched by no other T2DM treatment currently available. We now expect to learn from studies of new transgenic mice over-expressing COUP-TFII combined or not with GLP-1 analog treatment whether COUP-TFII will reduce and/or reverse the development of diabetes.

## Methods

### Mouse genetics and genotyping

The derivation of the pdx1^CRE/-^COUP-TFII^Fl/Fl^ mouse line has been previously reported [3] except for the fact that the Cre recombinase in this study is under the control of Pdx1 promoter (Pdx1-Cre transgenic line provided by Dr Melton, [25]). COUP-TFII PCR analysis was used to genotype mice using DNA isolated from tail tips of newborns as already described [3]. Male or female mutant mice were compared to COUP-TFII^Fl/Fl^ and pdx1^CRE/-^ littermates of the same age and sex. Embryos were studied at embryonic day (E) 12.5 and E14.5; newborns were studied at 1 day, 5 days and pups at 21 days after birth; adult mice were studied at 20 weeks of age. Animals were housed in colony cages with a 12-h light/12-h dark cycle in a temperature-controlled environment and fed ad libitum with a standard laboratory chow diet: composition 65% carbohydrate, 11% lipids and 24% proteins (SAFE, France R03-10). All procedures were performed in accordance with the principles and guidelines established by the European Convention for the Protection of Laboratory Animals under animal experimentation permit number 5820 from the Ministre de l'enseignement supérieur et de la recherché (France) and animal care facility agreement number A-75-14-02.

### Immunostaining and quantification of β-cell and α-cell mass in 21-day-old and adult mice

Pancreases were removed from sacrified animals and weighed. Whole pancreases were Bouin fixed and paraffin embedded. Each pancreatic block was serially sectioned (7 µm thickness) throughout its length. Every 35 sections throughout the block, ten sections were randomly chosen and immunostained for insulin or glucagon using indirect peroxidase labeling. Sections were incubated for 1 h with primary antibodies guinea pig anti-insulin serum (dilution 1∶1000) or rabbit antiglucagon serum (dilution 1∶600) from MP Biomedicals, Orsay, France. Sections were incubated for 45 min with peroxydase-conjugated rabbit anti-guinea pig IgG (dilution 1∶50; Dako, Trappes, France) or goat anti-rabbit IgG (final dilution 1∶50; Dako) then visualized with 3.39-diaminobenzidine-tetra-hydrochloride (DAB) (kit DAB; Valbiotech, Compiegne, France). Sections were mounted in Eukitt (Labonord).

For each section, the total surface area and area of β-cells (insulin-positive) or α-cells (glucagon-positive) were estimated by computer-assisted image analysis using an Olympus BH2 microscope connected via a color video camera to a personal computer with Histolab 5.14 software (Microvision Instruments, Evry, France). The relative volume of β- or α-cells was determined by stereological morphometric methods, calculating the ratio between the area occupied by immunoreactive cells and that occupied by all pancreatic cells. Total β-cell or α-cell mass per pancreas was calculated by multiplying this ratio by the whole pancreas mass.

COUP-TFII expression in adult pancreatic sections from COUP-TFII^Fl/Fl^ and pdx1^CRE/-^COUP-TFII^Fl/Fl^ was assessed as previously described [4].

### Estimation of endocrine cell number and mass in E12.5, E14.5 and newborn mice

Each pancreatic block was serially sectioned (8 µm thickness) throughout its length. From each pancreas from newborns, 1 section was quantified every 10 sections, giving a total of about 20 sections. For each E12.5 pancreas, 1 section was quantified every 3 sections, giving a total of about 12 sections. For each E14.5 pancreas, 1 section was quantified every 5 sections giving a total of about 12 sections. Sections were incubated overnight with primary antibodies guinea pig anti-insulin serum (dilution 1∶100, Dako) and rabbit anti-glucagon serum (dilution 1∶600, Linco). Sections were then incubated for 1 h with Alexa Fluor 488-conjugated anti-rabbit IgG (final dilution 1∶200; Molecular Probes) and Alexa Fluor 555-conjugated goat anti-guinea pig IgG (final dilution 1∶1000; Molecular probes). Slides were counterstained with DAPI (diamidino-2-phenylindole). Sections were mounted in PBS-glycerol (1∶1, v/v). E12.5 and E14.5 adjacent sections were stained overnight with rabbit anti-Pdx1 (1∶60,000, courtesy of Chris Wright). Then sections were incubated for 1 h with Alexa Fluor 488-conjugated anti-rabbit IgG (final dilution 1∶200; Molecular Probes). The total numbers of β-cells or α-cells at E12.5 and E14.5 were counted using a Leica DM5500 microscope connected via a color video camera to a personal computer although E12.5 insulin-positive cells were not numerous enough to be counted reliably. Pdx1-positive areas were quantified on mosaic images taken with the same microscope with a 20X objective using ImageJ sofware. Areas with signal intensity above a defined threshold were counted. To quantify the DAPI area, mosaic images were captured with the same microscope/camera/computer set-up with a 10× objective. Positive areas were defined with the lasso function and measured using Image J sofware.

### Histological studies

Five-day-old pancreases were removed from sacrificed animals (4 mice per group) and weighed. Whole pancreases were fixed 12 h in 4% paraformaldehyde and paraffin embedded. Each paraffin block containing the pancreases was serially sectioned throughout its length.

### β-cell proliferation

Ten sections were randomly chosen and were double stained for insulin and Ki67 (NCL-Ki67P, Leica). To estimate the proliferation rate, β-cells and Ki67-positive β-cells were counted using an Olympus BX40 microscope. At least 2,000 cells were counted per pancreas.

### β-cell apoptosis

Apoptotic cells were detected by terminal deoxynucleotidyl transferase dUTP-mediated nick-end labeling (TUNEL; Millipore) assay as previously described [7].

### β-cell neoformation from ductal precursors

To obtain an estimation of β-cell neogenesis activation, we quantified single β-cells and clusters of β-cells in the duct epithelium as previously described [22]. Values for insulin+/β-cells are related to total pancreatic section area and cells were determined per µm^2^ of pancreatic tissue at P5.

#### Intraperitoneal glucose tolerance test and insulin assay

Intraperitoneal glucose tolerance tests were performed on mice that had been fasted for 14 h. Animals were injected intraperitoneally with 2 g/kg body weight of glucose. Blood was withdrawn from the tail vein. Blood glucose was measured immediately before and 20, 40, 60, and 120 min after glucose injection using the Accu-Check II glucometer (Roche Diagnostic). Blood samples taken at the same times were immediately centrifuged at 4°C, and the plasma was stored at -80°C until assayed. Plasma insulin was measured with a radioimmunoassay kit (Diasorin, Antony, France).

#### Cell culture

The rat insulinoma 832/13 INS-1 cell line was used between passages 7 and 25 [49]. Cells were cultured at 5% CO_2_–95% air at 37°C in RPMI 640 medium containing 11 mM D-glucose supplemented with 10% (v/v) heat-inactivated fetal bovine serum, 100 U/ml penicillin-streptomycin, 10 mM HEPES, 1 mM sodium pyruvate (Invitrogen), and 50 µM β-mercaptoethanol (Invitrogen) (INS-1 medium). Cells in INS-1 medium were stimulated with 10 nM Exd4 (Exenatide, Byetta) or 100 nM GLP-1 (7-36) amide (Catalogue number 028-11, Phoenix) for 15 min or 6 h. Human islets were isolated from pancreases of three healthy organ donors as described previously [50], [51], [52] cultured in full CMRL medium (Invitrogen) [34] on extracellular matrix–coated plates derived from bovine corneal endothelial cells (Novamed Ltd., Jerusalem, Israel), allowing the cells to attach to the dishes and spread for 24 h before transfection to preserve their functional integrity [53], [54].

#### Gene silencing with siRNA

COUP-TFII and TCF7L2 (SI03385851, Qiagen) depletion experiments in the 832/13 INS-1 cell line were performed as described previously [5]. Cells (1.2×10^6^) were transfected with 68 and 50 pmol of COUP-TFII siRNA and TCF7L2 siRNA respectively, in parallel to control siRNA, by electroporation using an Amaxa Nucleofector II device (solution T, program T20; Amaxa Biosystems) and cultured during 48 h.

Isolated human islets were exposed to transfection _Ca_^2+^-KRH medium (4.74 mM KCl, 1.19 mM KH2PO4, 1.19 mM MgCl_2_6H_2_O, 119 mM NaCl, 2.54 mM CaCl2, 25 mM NaHCO3, 10 mM HEPES). SiRNA-Lipofectamine 2000 complexes were prepared according to the manufacturer's instructions (Lipofectamine2000; invitrogen) using 50 nM siRNA to TCF7L2 (RNAs of 21 nucleotides, designed to target human *TCF7L2*; Stealth Select^TM^ RNAi, invitrogen) and scrambled siRNA (Ambion, Austin, TE) as described before [55]. After overnight incubation, the transfection medium was aspirated and replaced by fresh culture medium. Islets were fixed 72 h later in Bouińs fixative embedded in paraffin and islet sections were prepared after dehydration. Sections were deparaffinized, rehydrated and incubated overnight at 4°C with mouse anti-COUP-TFII (Perseus Proteomics H7147-00) and guinea pig anti-insulin antibodies (Dako, Carpinteria, CA), followed by detection using cy3 and fluorescein-conjugated antibodies (Jackson) and embedded in Vectashield mounting medium (Vector Laboratories, Burlingame, CA), to visualize all cells by 4,6-diamidino-2-phenylindole (DAPI) staining. Fluorescence was analyzed using a Nikon MEA53200 (Nikon GmbH Duesseldorf, Germany) microscope and images were acquired using Openlab (Improvision Inc, Waltham, MA 02451,USA) or NIS-Elements software (Nikon). Western blotting was performed to check the level of downregulation of TCF7L2 and resulted in 4-fold downregulation compared to siScr-treated control as shown before [45].

#### Cell viability assay

After 48 h of COUP-TFII inactivation by siRNA, cell viability was assayed using the MTT (3-(4,5-dimethylthiazol-2-yl)-2,5-diphenyl tetrazolium bromide) assay following the Cellitoter 96 assay (Promega G4000) procedure.

#### Flow cytometry measurements

Cultured 832/13 INS-1 cells were pretreated with scrambled or COUP-TFII-specific siRNA for 48 h. Proliferation of β-cells was evaluated by measuring 5-bromo-2′-deoxyuridine (BrdU) incorporation using APC BrdU Flowkit (catalogue number 552598, BD Pharmingen). Cells were incubated with BrdU (10 µM) for 1 h at 37°C. Then cells were washed, trypsinized and fixed with cytofix/cytosperm buffer. Development of BrdU staining was done following the kit procedure. DNA was stained with 7-aminoactinomycin D (7-AAD) and cells were analyzed by using FACS Calibur4C (Becton Dickinson) with the FL-1 channel (band pass 530 ± 30 nm) for BrdU and the FL-3 channel (long pass 670 nm) for 7-ADD. Changes in mitochondrial membrane potential (Δψm) were evaluated by labeling cells with DiOC_6_(3). Cells were incubated for 20 min at room temperature with 10 nM DiOC_6_(3) and labeled with propidium iodide (PI, 1 mg/ml). Cell fluorescence was measured after suitable spectral compensation in the FL-1 channel for DiOC_6_(3) and the FL-3 channel for PI. To induce apoptosis, cells were treated with a rat cytokine mix containing 25 ng/ml TNF-α (catalogue number 400-14, Peprotech), 10 ng/ml IL-1β (catalogue number 400-01, Peprotech), and 10 ng/ml INF-γ (catalogue number 400-20, Peprotech) during 24 h.

#### Plasmid, transfection and reporter gene assay

The T-cell factor (TCF)-responsive TOPflash vector (Addgene) expressing luciferase driven by multiple TCF-responsive elements was used to evaluate the activity of β-catenin. Transfection and reporter gene assays were done as previously described [4].

#### Adenovirus infection

Recombinant adenoviruses expressing GFP alone (Ad-GFP) or with human COUP-TFII (Ad-hCOUP-TFII) were generated and used as described previously [4]. Recombinant adenovirus expressing GFP alone (Ad-GFP) or with human wild-type β-catenin (Ad-β catenin) were generously provided by Dr CJ Li [56]. After COUP-TFII gene silencing, β-cells were infected with a multiplicity of infection of 20 in 500 µ l INS-1 medium for 2 h at 37°C and then cultured for 24 h before stimulation with or without Exd4 for 6 h.

#### Pancreatic islet isolation

Pancreatic islets of 5 week-old-pdx1^CRE/-^, COUP-TFII^Fl/Fl^ and COUP-TFII^Fl/Fl^ pdx1^CRE/-^ mice were isolated as previously described [4]. Total RNA was extracted from hand-picked islets using the Absolutely RNA microprep kit according to the instructions provided by the manufacturer (Stratagen).

#### Microarray expression profiling and data analysis

We determined the RNA profile of COUP-TFII knockdown β-cells (832/13 INS-1 cells transfected with COUP-TFII specific siRNA) with respect to control cells (832/13 INS-1 cells transfected with scrambled siRNA) using Affymetrix expression analysis technical manual 701025Rev.5 (Affymetrix, Santa Clara, California, USA). Briefly, 1 mg of total cellular mRNA was reverse transcribed into cDNA (SuperScript Choice System Invitrogen, Carlsbad, CA) using oligo-dT primers and a T7 RNA polymerase promoter site. The cDNA was *in vitro* transcribed and biotin-labeled for microarray analysis using the Affymetrix IVT labeling kit. The concentration of labelled cRNA was measured using a NanoDrop ND-1000 spectrophotometer. Labeled cRNA was fragmented in a fragmentation buffer for 35 min at 94°C. The quality of labeled and fragmented cRNA was analyzed using the Agilent bioanalyzer 2100 [57]. Fragmented cRNA was hybridized to the rat 230 2.0 array (Affymetrix) during 16h at 45°C. Arrays were washed and stained in a fluidics station (Affymetrix) and scanned using the Affymetrix 3000 GeneScanner.

Intensity of microarray probe signals were background-subtracted and normalized using reference probes. Individual probe values from 3 chips per treatment were used to calculate mean fold change. Log2-transformed values were employed for statistical analysis (Student's *t* test, Figure S3). Significant changes in gene expression (where *P*≤0.05) were filtered, annotated using NetAffx Data Analyses Center v31 [58]; http://www.affymetrix.com/analysis/index.affx) and analyzed using Ingenuity Pathway Analysis (Ingenuity® Systems; www.ingenuity.com), Gene Set Analysis Toolkit V2 (GSAT; [59] and David Bioinformatics Resources 6.7 (DavidBR, [60]. Results are available in the GEO database, accession number GSE30526 and are shown in Figure S2 and Figure S3.

#### Isolation of total mRNA and transcript detection by RT-qPCR

Total RNA was extracted and purified from cultured cells using the RNA-Plus reagent according to the manufacturer's institutions (Q-BIOgene). Reverse transcription was done with 2 µg of total RNA using Superscript II reverse transcriptase (Invitrogen) according to the manufacturer's protocol. RT-qPCR was performed with 6.25 ng of reverse-transcribed total RNA, 10 µM of each primer (Eurogentec), and 2 mM MgCl_2_ in 1x LightCycler DNA Master SYBR Green I mix using a LightCycler device (Roche). All samples were normalized to the threshold cycle value for cyclophilin mRNA, chosen as an invariant control. Forward and reverse primers used for the specific amplification of cDNA fragments were described by [5] or designed to hybridize to rat and/or mouse transcripts. Primer sequences will be provided on request.

Islet cDNA was prepared from GipR^-/-^; Glp-1-R^-/-^ transgenic mice and control littermates [20] and gene expression analysis was performed by RT-qPCR as above.

#### Immunoblotting

Total and cytosolic 832/13 INS-1 proteins were prepared as described in reference [4]. Protein extracts were sonicated and 10 to 50 µg was subjected to SDS-PAGE followed by immunoblotting with specific antibodies for: cyclin D1 at 1∶1,000 dilution (Neomarkers clone sp4 RM-9104/monoclonal rabbit anti-human) ; CDK4 at 1∶1,000 dilution (catalogue number 6315, Abcam); β-actin at 1∶2,000 dilution (catalogue number A2066, Sigma) or unphosphorylated (active) β-catenin at dilution 1∶1,000 dilution (catalogue number 05-665, Millipore). Immunoreactive bands were revealed using ECL SuperSignal West Pico chemiluminescent reagents (Pierce). Autoradiograms were scanned using Chemi Genius 2 Bioimaging System and quantified using Image J software.

#### Statistical analysis

Quantitative results are expressed as means ± standard errors of the mean (SEM). Statistical analyses were carried out using the Mann-Whitney test, a nonparametric statistical program appropriate when the sample number is less than 10. Null hypotheses were rejected at *P* values of >0.05. All experiments were performed at least three times.

## Supporting Information

Figure S1 **Absence of COUP-TFII in mouse pancreatic β-cells does not modify α-cell number**.
(TIF)

Figure S2 **Categorization of COUP-TFII modulated genes in 832/13 INS-1 cells by biological process, molecular function and cellular component.**
(PDF)

Figure S3 **Analysis of broad gene ontology terms (GO Slim) based on GSAT v2.**
(PDF)

Figure S4 **Exd4 stabilizes cytosolic endogenous and over-expressed β-catenin in 832/13 INS-1 β-cells and Exd4 induces cyclin D1 mRNA levels via β-catenin signaling pathway.**
(TIF)

Figure S5 **COUP-TFII expression in mouse ductal epithelium and in periductal mesenchyme at 18.5.**
(TIF)
