# Supplementary figures and images for: COUP-TFII Controls Mouse Pancreatic β-Cell Mass through GLP-1-β-Catenin Signaling Pathways
Source: PLoS One. 2012 Jan 24;7(1):e30847. doi: 10.1371/journal.pone.0030847 (PMC3265526; doi:10.1371/journal.pone.0030847)

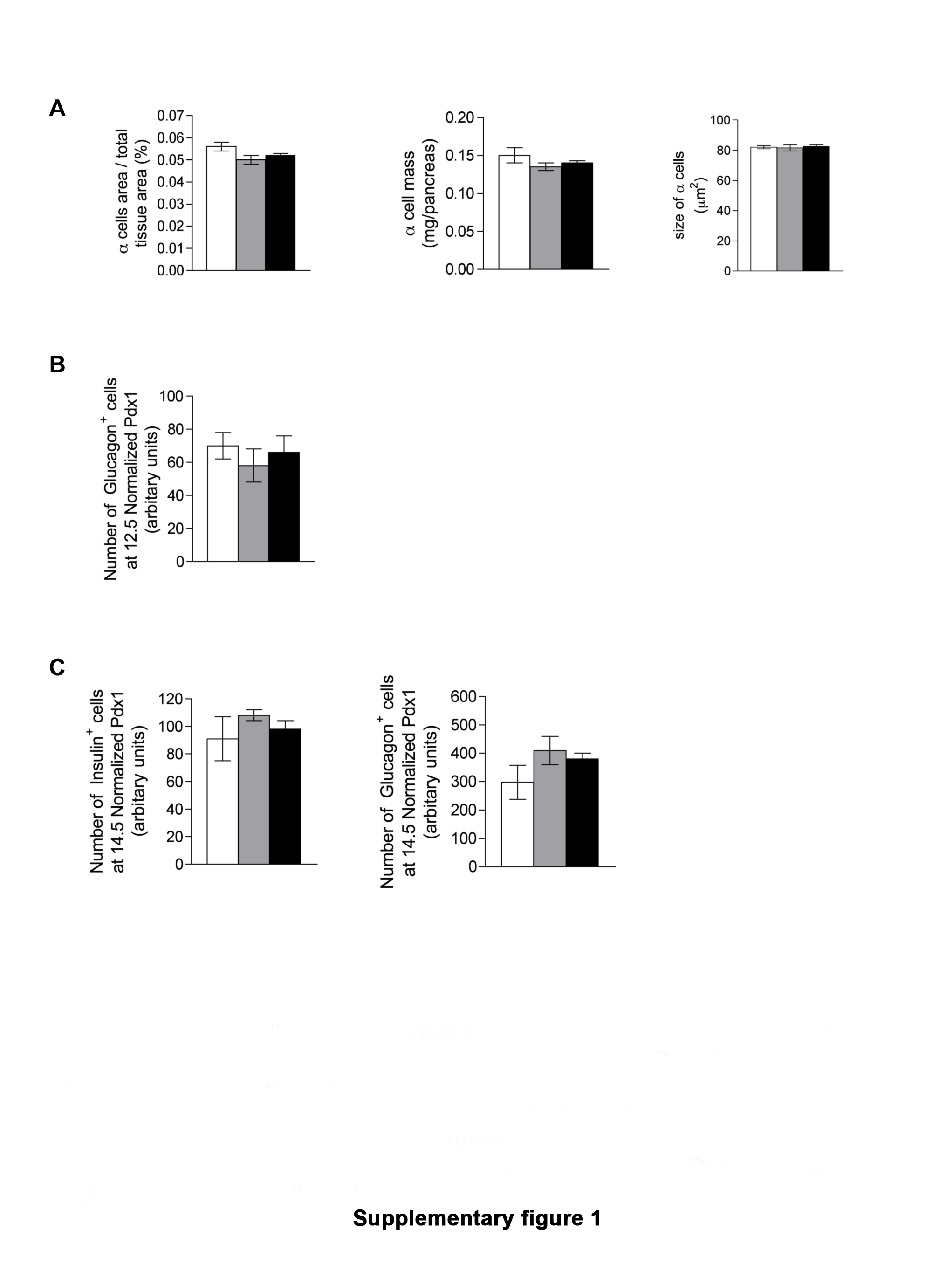

Supplement: Figure S1 — Absence of COUP-TFII in mouse pancreatic β-cells does not modify α-cell number. (TIF) [file pone.0030847.s001.tif]

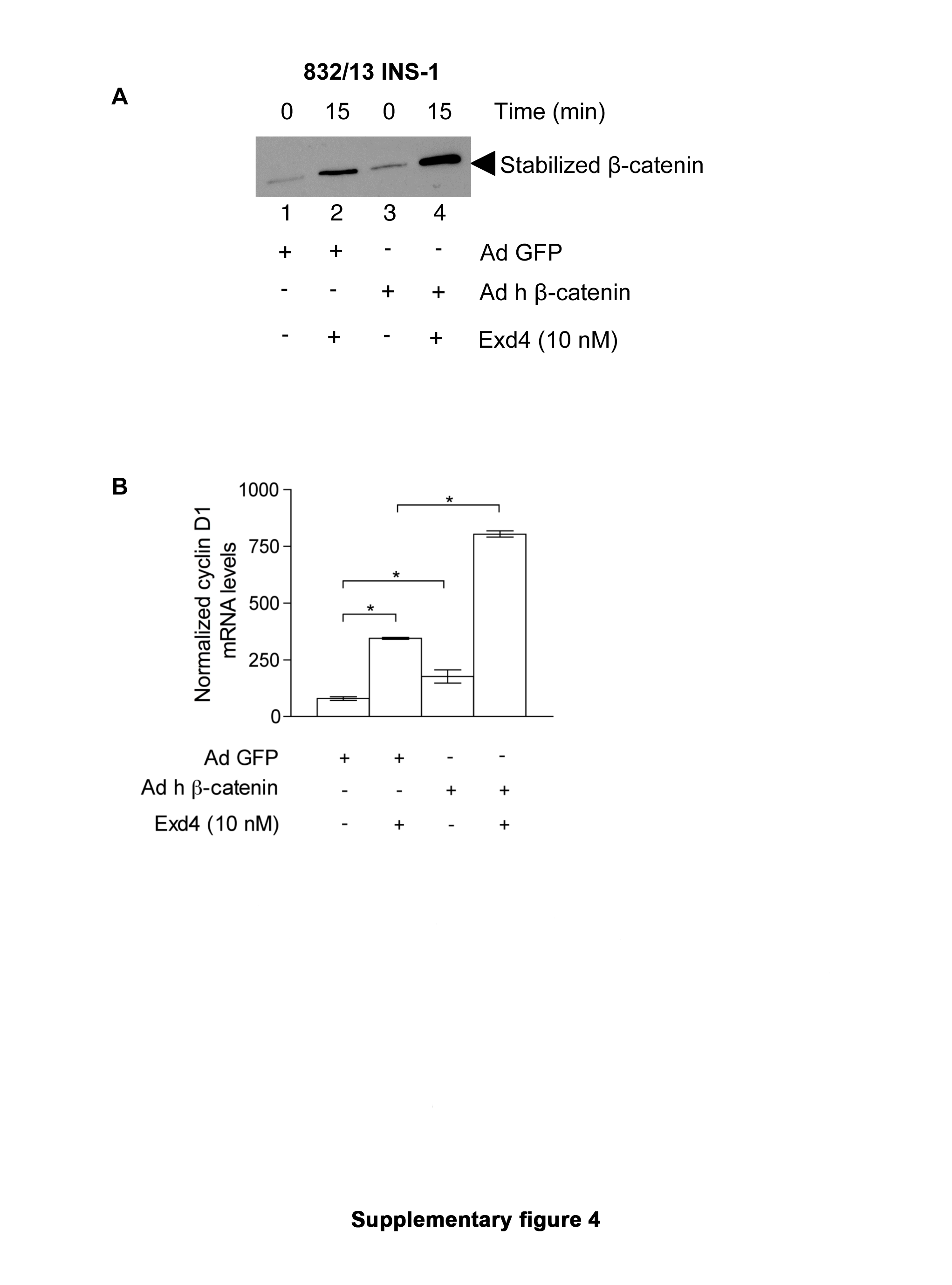

Supplement: Figure S4 — Exd4 stabilizes cytosolic endogenous and over-expressed β-catenin in 832/13 INS-1 β-cells and Exd4 induces cyclin D1 mRNA levels via β-catenin signaling pathway. (TIF) [file pone.0030847.s004.tif]

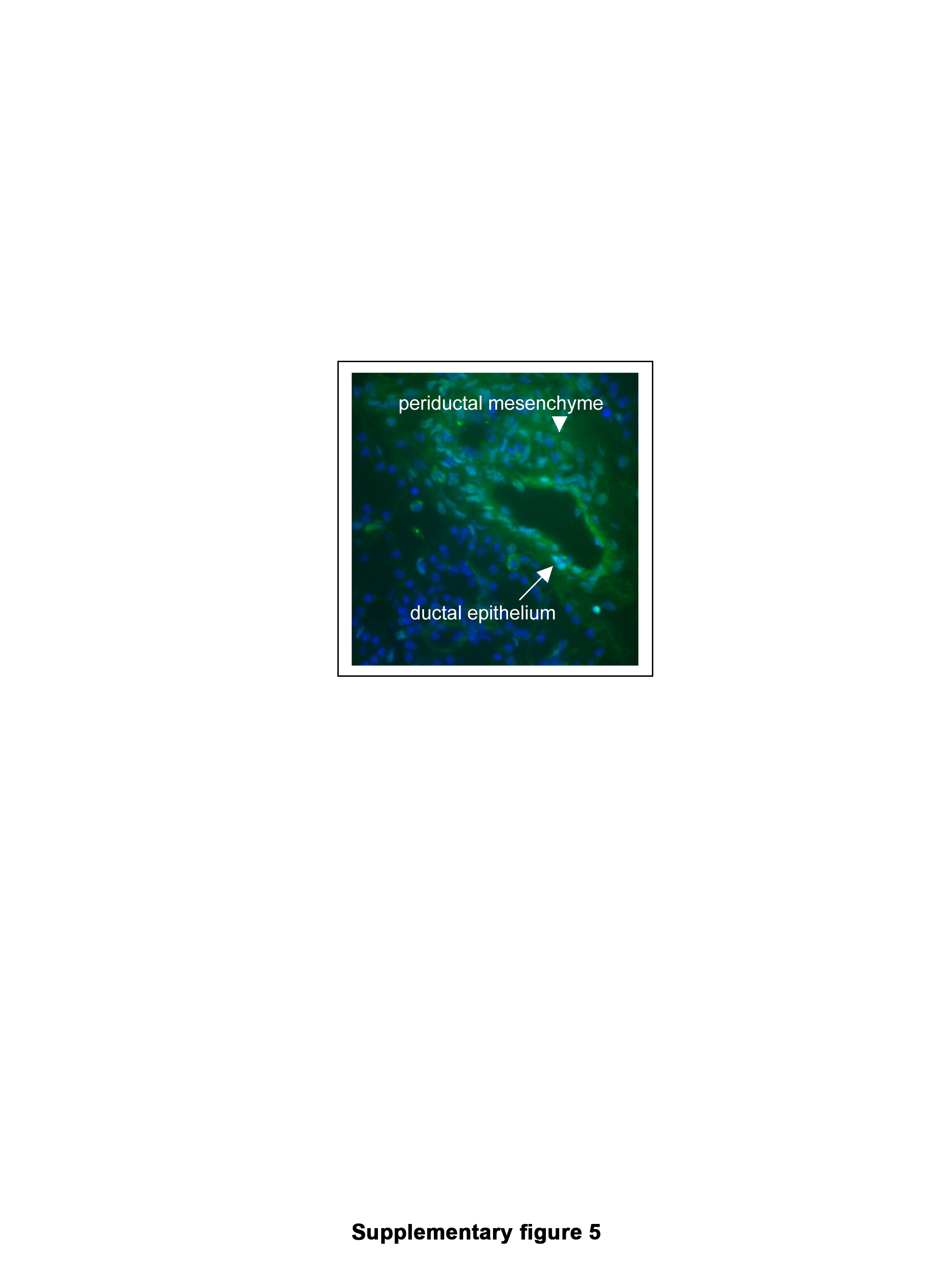

Supplement: Figure S5 — COUP-TFII expression in mouse ductal epithelium and in periductal mesenchyme at 18.5. (TIF) [file pone.0030847.s005.tif]
