# Supplementary material for: COUP-TFII Controls Mouse Pancreatic β-Cell Mass through GLP-1-β-Catenin Signaling Pathways
Source: PLoS One. 2012 Jan 24;7(1):e30847. doi: 10.1371/journal.pone.0030847 (PMC3265526; doi:10.1371/journal.pone.0030847)
Supplement: Figure S2 — Categorization of COUP-TFII modulated genes in 832/13 INS-1 cells by biological process, molecular function and cellular component. (PDF) [file pone.0030847.s002.pdf]

Supporting fig 2. Categorization of COUP-TFII modulated genes in 832/13 INS-1 cells by biological process, molecular function and cellular component.

Biological process		Transcripts	% of transcripts
All		587	100,00
Metabolic process		231	39,35
Biological regulation		227	38,67
Multicellular organismal process		128	21,81
Cell communication		121	20,61
Localization		109	18,57
Response to stimulus		103	17,55
Developemental process		96	16,35
Cellular compartiment organization		76	12,95
Cell proliferation		38	6,47
Death		31	5,28
Reproduction		27	4,60
Growth		15	2,56
Multi-organism process		13	2,21
Unclassified		221	37,65
	Sum	1436	244,63

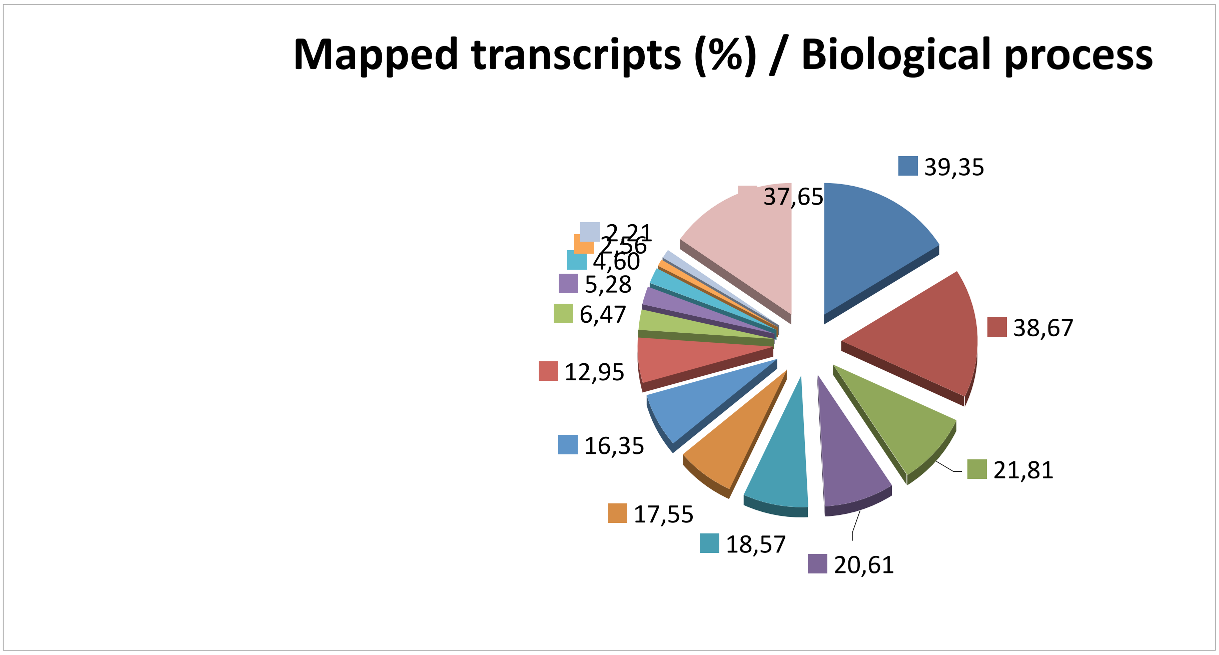

Molecular Function	Transcripts	% of transcripts
All	587	100,00
Protein binding	274	46,68
Ion binding	91	15,50
Nucleic acid binding	70	11,93
Nucleotide binding	56	9,54
Hydrolase activity	55	9,37
Molecular transducer activity	48	8,18
Transferase activity	44	7,50
Transporter activity	36	6,13
Enzyme regulator activity	36	6,13
Structural molecule activity	30	5,11
Transcription regulator activity	17	2,90
Lipid binding	12	2,04
Carbohydrate binding	9	1,53
Electron carrier activity	7	1,19
Chromatin binding	5	0,85
Molecular adaptor activity	4	0,68
Translation regulator activity	3	0,51
Antioxidant activity	1	0,17
Oxygen binding	1	0,17
Unclassified	168	28,62

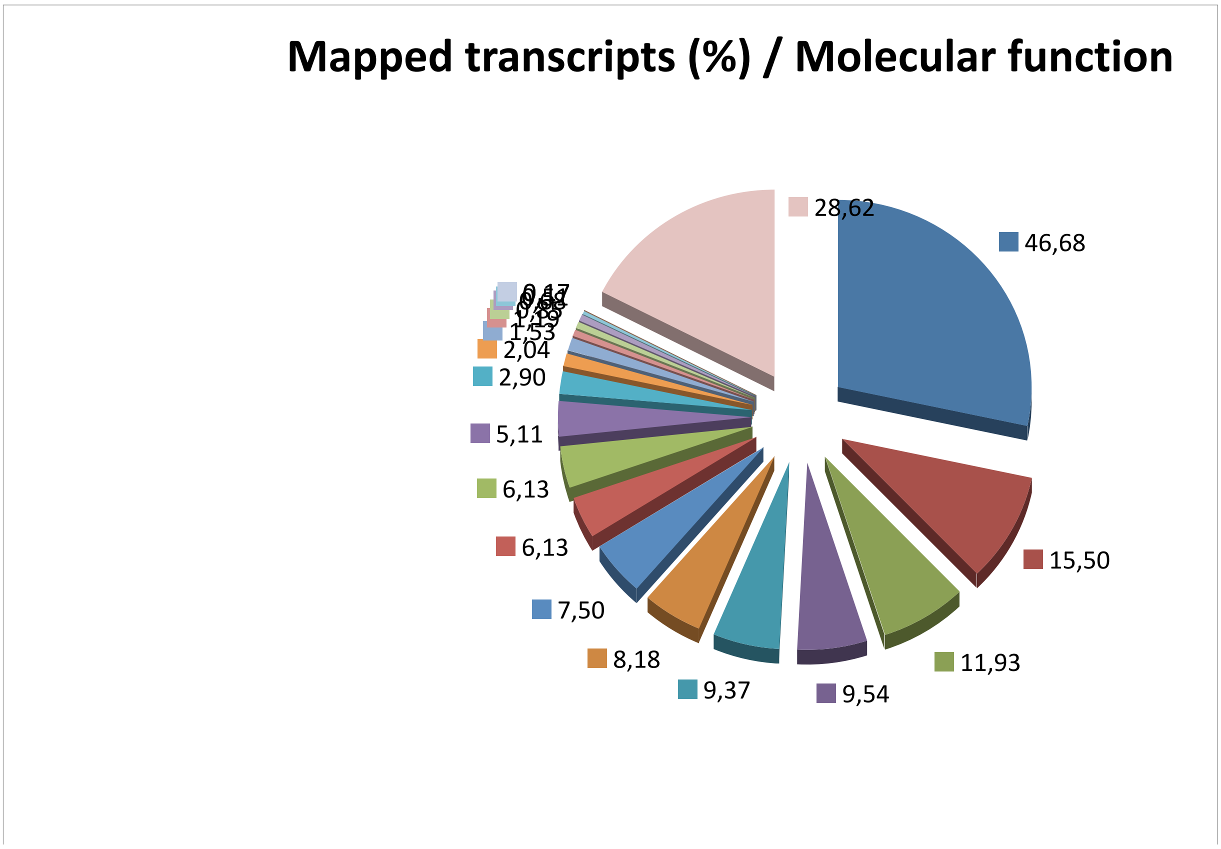

Cellular component	Transcripts	% of transcripts
All	587	100,00
Membrane	168	28,62
Nucleus	120	20,44
Macromolecular complex	76	12,95
Membrane-enclosed lumen	52	8,86
Cytosol	45	7,67
Cell projection	36	6,13
Mitochondrion	36	6,13
Golgi apparatus	34	5,79
Endoplasmic reticulum	29	4,94
Extracellular space	28	4,77
Cytoskeleton	25	4,26
Vesicle	24	4,09
Endomembrane system	23	3,92
Endosome	14	2,39
Vacuole	13	2,21
Extracellular matrix	11	1,87
Envelope	11	1,87
Chromossome	9	1,53
Ribossome	6	1,02
Microbody	2	0,34
Lipid particle	1	0,17
Unclassified	235	40,03

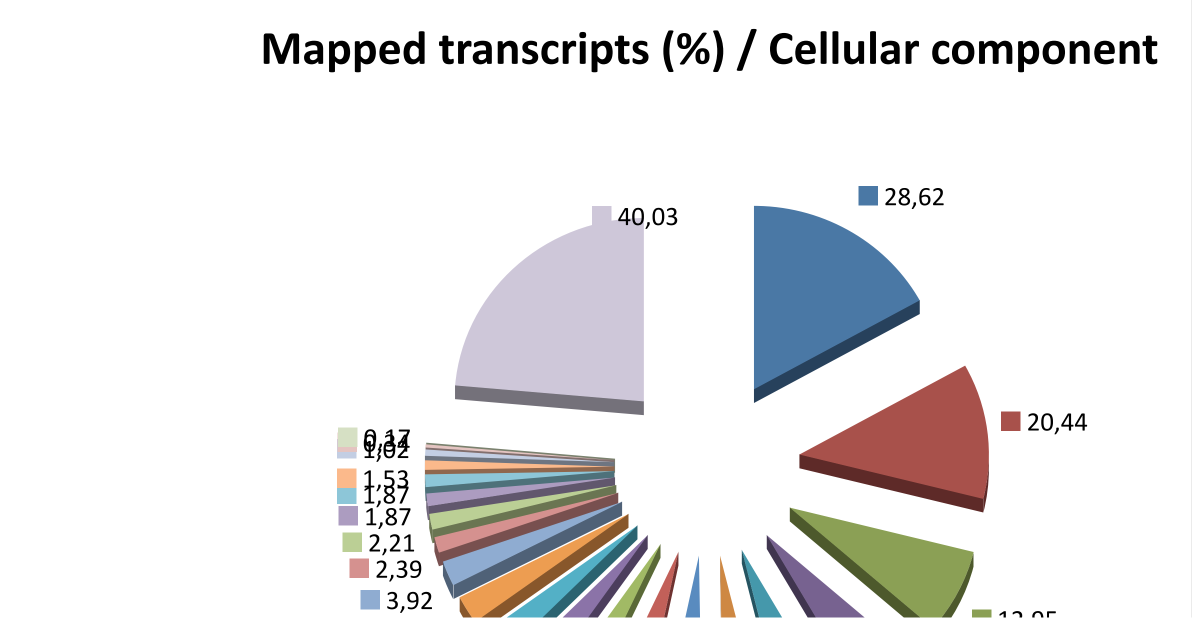

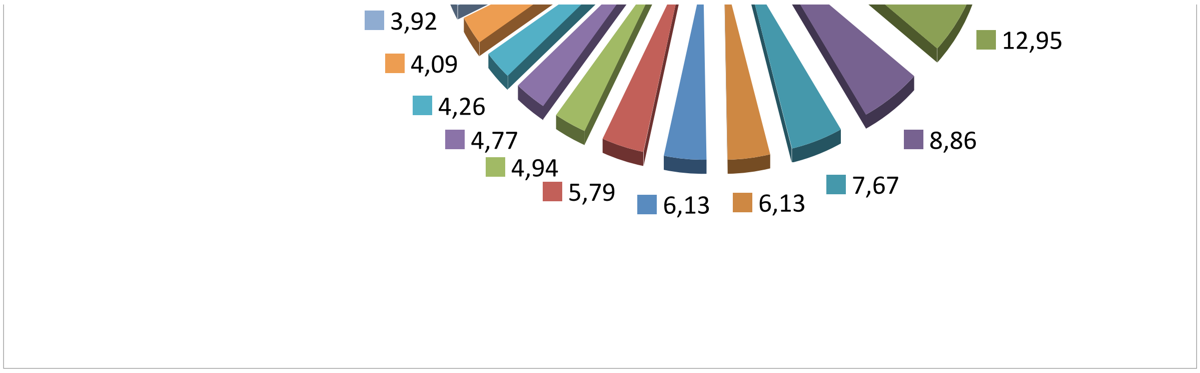
3,92
4,09
4,26
4,77
4,94
5,79
6,13
6,13
7,67
8,86
12,95
