# Supplementary material for: COUP-TFII Controls Mouse Pancreatic β-Cell Mass through GLP-1-β-Catenin Signaling Pathways
Source: PLoS One. 2012 Jan 24;7(1):e30847. doi: 10.1371/journal.pone.0030847 (PMC3265526; doi:10.1371/journal.pone.0030847)
Supplement: Figure S3 — Analysis of broad gene ontology terms (GO Slim) based on GSAT v2. (PDF) [file pone.0030847.s003.pdf]

**Supporting fig 3. Analysis of broad gene ontology terms (GO Slim) based on GSAT v2. Genes associated to selected pathways and processes that are probably involved in the consequences of COUP-TFII modulation are listed below with their respective fold change and the statistical significance of their variations.**

## (a) Wnt_pathway

| | |
|---|---|
| **KEEG** | Wnt signaling pathway |
| **GO** | regulation of Wnt receptor signaling pathway (0030111) |

| Probe set ID | Symbol | Name | Fold change | Unpaired t-test |
|---|---|---|---|---|
| 1388187_at | Camk2a | calcium/calmodulin-dependent protein kinase II alpha | -1,87 | 4,5E-03 |
| 1374392_at | Csnk1a1 | casein kinase 1, alpha 1 | 1,07 | 8,2E-03 |
| 1378282_at | Csnk2a2 | casein kinase 2, alpha prime polypeptide | -1,52 | 1,1E-02 |
| 1382160_at | Csnk2a2 | casein kinase 2, alpha prime polypeptide | -1,41 | 1,4E-02 |
| 1388774_at | Mbd2 | methyl-CpG binding domain protein 2 | -1,21 | 1,6E-02 |
| 1383075_at | Ccnd1 | cyclin D1 | -2,36 | 2,1E-02 |
| 1375961_at | Frzb | frizzled-related protein | -1,70 | 2,8E-02 |
| 1379237_at | Tax1bp3 | Tax1 (human T-cell leukemia virus type I) binding protein 3 | -3,97 | 3,2E-02 |
| 1374175_at | Porcn | porcupine homolog (Drosophila) | 1,47 | 3,3E-02 |
| 1388965_at | Ppp2r5e | protein phosphatase 2, regulatory subunit B', epsilon isoform | -1,21 | 3,3E-02 |
| 1388392_at | Tax1bp3 | Tax1 (human T-cell leukemia virus type I) binding protein 3 | 1,13 | 3,4E-02 |
| 1376616_at | Csnk2a1 | casein kinase 2, alpha 1 polypeptide | -1,05 | 3,8E-02 |
| 1380023_at | SENP2 | SUMO1/sentrin/SMT3 specific peptidase 2; similar to SUMO/sentrin speci | -1,34 | 3,9E-02 |
| 1379942_at | Sox2 | SRY (sex determining region Y)-box 2 | 3,50 | 4,4E-02 |
| 1397754_at | Wwp1 | WW domain containing E3 ubiquitin protein ligase 1 | -3,27 | 4,5E-02 |
| 1395502_at | Ppp2r5d | protein phosphatase 2, regulatory subunit B', delta isoform | 1,46 | 4,7E-02 |
| 1374654_at | Btrc | beta-transducin repeat containing | -1,20 | 5,0E-02 |
| 1394444_at | Fbxw11 | F-box and WD repeat domain containing 11 | -3,29 | 5,0E-02 |

## (b) Insulin_pathway

**KEEG** Insulin signaling pathway

**Ingenuity** Insulin receptor signaling

| Probe set ID | Symbol | Name | Fold change | Unpaired t-test |
|---|---|---|---|---|
| 1370893_at | Acaca | acetyl-coenzyme A carboxylase alpha | -1,29 | 6,7E-03 |
| 1383277_at | Ppp1r7 | protein phosphatase 1, regulatory (inhibitor) subunit 7 | 1,27 | 1,3E-02 |
| 1375213_at | Pck2 | phosphoenolpyruvate carboxykinase 2 (mitochondrial) | -1,36 | 1,7E-02 |
| 1388965_at | Ppp2r5e | protein phosphatase 2, regulatory subunit B', epsilon isoform | -1,21 | 3,3E-02 |
| 1368370_at | Adcy4 | adenylate cyclase 4 | 2,75 | 3,5E-02 |
| 1369912_at | Crk | v-crk sarcoma virus CT10 oncogene homolog (avian) | 1,83 | 3,6E-02 |
| 1394384_at | Drd4 | dopamine receptor D4 | -3,05 | 3,8E-02 |
| 1369271_at | Prkab2 | protein kinase, AMP-activated, beta 2 non-catalytic subunit | 2,82 | 4,0E-02 |
| 1369577_at | Socs2 | suppressor of cytokine signaling 2 | 1,22 | 4,0E-02 |
| 1368826_at | Comt | catechol-O-methyltransferase | 1,31 | 4,1E-02 |
| 1393402_at | Crkl | v-crk sarcoma virus CT10 oncogene homolog (avian)-like | -1,21 | 4,6E-02 |
| 1395502_at | Ppp2r5d | protein phosphatase 2, regulatory subunit B', delta | 1,46 | 4,7E-02 |
| 1385959_at | Sos1 | Son of sevenless homolog 1 | -1,28 | 4,7E-02 |
| 1368605_at | Sh2b2 | SH2B adaptor protein 2 | -1,17 | 4,9E-02 |

## (c) Lipid

**GO** cellular lipid metabolic process (0044255)

**FABP9** Lipid binding (GO: 0008289)

| Probe set ID | Symbol | Name | Fold change | Unpaired t-test |
|---|---|---|---|---|
| 1387748_at | Lep | Leptin | 5,62 | 1,8E-03 |
| 1374440_at | Hsd17b11 | Hydroxysteroid (17-beta) dehydrogenase 11 | -1,42 | 2,8E-03 |
| 1384136_at | Osbpl3 | Oxysterol binding protein-like 3 | 1,20 | 5,5E-03 |
| 1388210_at | Acot2 | Acyl-CoA thioesterase 2 | -1,26 | 6,5E-03 |
| 1372919_at | Agpat6 | 1-acylglycerol-3-phosphate O-acyltransferase 6 (lysophosphatidic acid ac | 1,12 | 7,0E-03 |
| 1375600_at | Pigo | Phosphatidylinositol glycan anchor biosynthesis, class O | -1,34 | 7,8E-03 |
| 1380834_at | Btnl8 | Butyrophilin-like 8 | -10,78 | 7,9E-03 |
| 1368630_at | Fabp9 | fatty acid binding protein 9, testis | -9,60 | 1,1E-02 |
| 1387156_at | Hsd17b2 | Hydroxysteroid (17-beta) dehydrogenase 2 | 2,39 | 1,4E-02 |
| 1384664_at | Sftpb | Surfactant associated protein B | 2,26 | 1,7E-02 |
| 1387423_at | Lhcgr | Luteinizing hormone/choriogonadotropin receptor | 2,69 | 2,2E-02 |
| 1384667_x_at | Galr2 | Galanin receptor 2 | -1,27 | 2,4E-02 |
| 1371137_at | Acox2 | Acyl-Coenzyme A oxidase 2, branched chain | 3,79 | 2,6E-02 |
| 1367702_at | Acadm | Acyl-Coenzyme A dehydrogenase, medium chain | -1,54 | 2,7E-02 |
| 1379111_at | Yy1 | YY1 transcription factor | 10,94 | 2,8E-02 |
| 1371923_at | Lpcat1 | Lysophosphatidylcholine acyltransferase 1 | -1,37 | 3,3E-02 |
| 1398147_at | Dpagt1 | Dolichyl-phosphate (UDP-N-acetylglucosamine) acetylglucosaminephosp | -3,35 | 3,9E-02 |
| 1369271_at | Prkab2 | Protein kinase, AMP-activated, beta 2 non-catalytic subunit | 2,82 | 4,0E-02 |
| 1374308_at | Sec14l2 | SEC14-like 2 (S. cerevisiae) | 1,28 | 4,0E-02 |
| 1368826_at | Comt | Catechol-O-methyltransferase | 1,31 | 4,1E-02 |
| 1369655_at | Pik3c3 | Phosphoinositide-3-kinase, class 3 | -1,40 | 4,1E-02 |

| | | | | |
|---|---|---|---|---|
| 1369701_at | Lipc | Lipase, hepatic | -5,98 | 4,3E-02 |
| 1367679_at | Cd74 | CD74 antigen (invariant polypeptide of major histocompatibility complex, c | 1,33 | 4,3E-02 |
| 1372663_at | Ptdss2 | Phosphatidylserine synthase 2 | 1,21 | 4,7E-02 |
| 1387037_at | Cubn | Cubilin (intrinsic factor-cobalamin receptor) | -6,91 | 4,8E-02 |
| 1370561_at | A3galt2 | Alpha 1,3-galactosyltransferase 2 (isoglobotriaosylceramide synthase) | 1,33 | 4,8E-02 |

## (d) Carbohydrate

**GO** cellular carbohydrate metabolic process (0044262)

**GO** negative regulation of gluconeogenesis (0045721)

| Probe set ID | Symbol | Name | Fold change | Unpaired t-test |
|---|---|---|---|---|
| 1387748_at | Lep | Leptin | 5,62 | 1,8E-03 |
| 1368836_a_at | Acan | Aggrecan | 5,18 | 1,9E-03 |
| 1386917_at | Pcx | Pyruvate carboxylase | -1,25 | 1,6E-02 |
| 1375213_at | Pck2 | Phosphoenolpyruvate carboxykinase 2 (mitochondrial) | -1,36 | 1,7E-02 |
| 1377264_at | Il17f | Interleukin 17F | 8,25 | 2,2E-02 |
| 1393828_at | Chid1 | Chitinase domain containing 1 | 4,53 | 2,2E-02 |
| 1387691_at | Tnf | Tumor necrosis factor (TNF superfamily, member 2) | 3,99 | 2,7E-02 |
| 1394334_at | Chst8 | Carbohydrate (N-acetylgalactosamine 4-0) sulfotransferase 8 | -1,28 | 3,1E-02 |
| 1398147_at | Dpagt1 | Dolichyl-phosphate (UDP-N-acetylglucosamine) acetylglucosaminephosp | -3,35 | 3,9E-02 |
| 1369701_at | Lipc | Lipase, hepatic | -5,98 | 4,3E-02 |
| 1369191_at | Il6 | interleukin 6 | 6,50 | 4,3E-02 |
| 1387920_at | Man2c1 | Mannosidase, alpha, class 2C, member 1 | 1,28 | 4,8E-02 |
